# Different Types of Laughter Modulate Connectivity within Distinct Parts of the Laughter Perception Network

**DOI:** 10.1371/journal.pone.0063441

**Published:** 2013-05-08

**Authors:** Dirk Wildgruber, Diana P. Szameitat, Thomas Ethofer, Carolin Brück, Kai Alter, Wolfgang Grodd, Benjamin Kreifelts

**Affiliations:** 1 Department of Psychiatry and Psychotherapy, Eberhard Karls University of Tübingen, Tübingen, Germany; 2 Institute of Neuroscience, Newcastle University, Newcastle upon Tyne, United Kingdom; 3 Department for Biomedical Magnetic Resonance, Eberhard Karls University of Tübingen, Tübingen, Germany; 4 Department of Psychiatry and Psychotherapy, University of Aachen, Aachen, Germany; University of Pennsylvania, United States of America

## Abstract

Laughter is an ancient signal of social communication among humans and non-human primates. Laughter types with complex social functions (e.g., taunt and joy) presumably evolved from the unequivocal and reflex-like social bonding signal of tickling laughter already present in non-human primates. Here, we investigated the modulations of cerebral connectivity associated with different laughter types as well as the effects of attention shifts between implicit and explicit processing of social information conveyed by laughter using functional magnetic resonance imaging (fMRI). Complex social laughter types and tickling laughter were found to modulate connectivity in two distinguishable but partially overlapping parts of the laughter perception network irrespective of task instructions. Connectivity changes, presumably related to the higher acoustic complexity of tickling laughter, occurred between areas in the prefrontal cortex and the auditory association cortex, potentially reflecting higher demands on acoustic analysis associated with increased information load on auditory attention, working memory, evaluation and response selection processes. In contrast, the higher degree of socio-relational information in complex social laughter types was linked to increases of connectivity between auditory association cortices, the right dorsolateral prefrontal cortex and brain areas associated with mentalizing as well as areas in the visual associative cortex. These modulations might reflect automatic analysis of acoustic features, attention direction to informative aspects of the laughter signal and the retention of those in working memory during evaluation processes. These processes may be associated with visual imagery supporting the formation of inferences on the intentions of our social counterparts. Here, the right dorsolateral precentral cortex appears as a network node potentially linking the functions of auditory and visual associative sensory cortices with those of the mentalizing-associated anterior mediofrontal cortex during the decoding of social information in laughter.

## Introduction

Laughter is an evolutionary old communication signal with high relevance for social interactions [1]. Tickling laughter is thought to be a more reflex-like behavior confined to the context of tickling and play which enforces play behavior and social bonding [2]. This laughter type is already present in non-human primates [3]. In humans, laughter has diversified beyond the primordial reflex-like laughter which is induced by tickling or play and which is related to play maintenance [4] and encompasses laughter types with both more complex social functions and positive as well as negative connotations (e.g., joy or taunt). The term “complex social laughter” refers to the fact that, in contrast to tickling laughter, these laughter types are produced in a wide variety of social situations and can be used in a conscious and goal-directed manner to influence and modify the attitudes and behaviors of our social counterparts [5], [6].

In a previous report based on the same fMRI data set as the present study and focusing on temporal and frontal brain regions [7], we delineated brain areas associated with the perception of these presumably evolutionary different laughter types. Complex social laughter types (CSL, i.e., joyful and taunting laughter) which were termed “emotional” laughter types in our previous report [7] elicited stronger cerebral responses in the anterior rostral mediofrontal cortex (arMFC) known to be activated during mentalizing tasks (i.e., inferring states of minds or intentions, [8]). Tickling laughter, in contrast, led to a stronger activation of the auditory association cortex presumably reflecting the higher acoustic complexity of the rapid and high-pitched tickling laughter [9] (see also Table S1). Similar activations of the auditory cortex have been described in connection with the perception of affective vocalizations including laughter [5], [10]–[13] and were found to be stronger for laughter as compared to speech [14]. In the neighboring research area of emotional prosody perception, stronger activations for emotional as compared to neutral speech melody have been demonstrated to be significantly associated with acoustic complexity [15]. Additionally, task-related focusing on the social information in the laughter signal increased activation in the orbitolateral part of the inferior frontal gyrus (olIFG) as well as the posterior rostral mediofrontal cortex (prMFC). As previous functional brain imaging studies on task-related effects during laughter perception were restricted to the perisylvian cortex, insula and amygdala [11], [12] and did not report task-related activation changes in these brain regions, the results of our previous study were discussed in relation to task-induced effects in studies on the perception of other signals of nonverbal vocal communication of emotional information: Activations in the olIFG seem to reflect explicit evaluation of social information in the nonverbal vocal signal parallel to neuroimaging studies on perception of emotional speech melody [16]–[22], attention direction to emotional prosody [23], working memory for prosodic cues [24], [25] and retrieval of memories associated with informative acoustic cues [26], [27]. PrMFC activation, on the other hand, appears consistent with the association of this region with focusing of attention and action monitoring [8], [28]–[31].

Recently, the notion that the neural substrates of cognitive functions in health and disease are also reflected in dynamic changes of connectivity between distinct and often distant brain regions has been supported by a fast growing amount of empirical evidence [32], [33]. In the area of speech comprehension and production, first attempts have been made to delineate patterns of brain connectivity underlying these cognitive functions [34]. With regard to non-verbal vocal cues (e.g., laughter or speech melody) available data is scarce: Ethofer and colleagues found evidence for a parallel flow of information within regions sensitive to explicit evaluation of emotional prosody from the right posterior temporal cortex to the bilateral olIFG using dynamic causal modeling [19]. In a recent study, Leitman and colleagues [35] described a frontotemporal network for processing of emotional prosody where cue saliency inversely modulated connectivity between the right IFG and the auditory processing regions in the right middle/posterior superior temporal cortex. With respect to the perception of laughter, to our knowledge only one study of brain connectivity [36] has been performed previously. Here, laughter and crying were used as nonverbal affective stimuli in contrast to control sounds. No previous study, however, addressed different types of laughter specifically.

Therefore, it was the aim of the present fMRI study to investigate modulations of neural connectivity between brain regions engaged in the perception of different types of laughter (i.e., joyful, taunting and tickling) to further elucidate the underpinnings of the neural processing of different aspects of the laughter signal (i.e., complexity of socio-relational content and acoustic complexity) and of different states of attention with regard to the social information carried in the laughter signal employing psycho-physiological interaction (PPI) analyses [37], [38]. Attention allocation towards or away from social information in laughter was modulated by two different tasks (i.e., laughter type categorization and laughter bout counting).

Based on the presently sole pertinent PPI analysis by Leitman and colleagues [35], we cautiously hypothesized that the lower degree of complex social information of tickling laughter, when interpreted as a lower degree of cue saliency when compared to CSL, would be associated with stronger connectivity between the right IFG and the right middle/posterior superior temporal gyrus (STG). A second tentative hypothesis was based on the study of Ethofer and colleagues [19] demonstrating flow of information among regions with stronger responses during explicit evaluation of emotional prosody. As the increased responses during laughter type categorization observed in the right pSTS and bilateral olIFG in our previous analysis [7] bear a striking resemblance to the activations observed by Ethofer and colleagues, we hypothesized that the explicit evaluation of social information in laughter would increase the connectivity between the right pSTS and bilateral olIFG. Finally, based on previous research indicating activation of the bilateral amygdalae through laughter [11]–[13], we defined this region as an additional region of interest for our analyses of hemodynamic activation and connectivity.

## Materials and Methods

### Participants

18 right-handed participants (9 m, 9 f, mean age ± SD: 26.0 years ±3.4 years) were included in the study. Handedness was assessed using the Edinburgh inventory [39]. None of the participants had a history of neurological or psychiatric illness, of substance abuse, of impaired hearing, or was on any medication. Vision was normal or corrected to normal in all participants.

### Ethics Statement

The study was approved by the University of Tübingen ethical review board and was performed in accordance with the Declaration of Helsinki. All participants gave written informed consent according to the guidelines of the University of Tübingen ethical review board prior to their inclusion in the present study.

### Stimulus Material

Laughter sequences portraying three types of laughter (joy (JOY), taunt (TAU), tickling (TIC); see Sounds S1, S2, and S3 for exemplars) served as stimulus material. The laughter sequences were produced by professional actors using an auto induction method based on an example scenario describing a situation of social communication [40]. For each type of laughter the actors were provided with one example scenario. In an independent behavioral study it was ascertained that all stimuli included in the present study could be identified well above chance level [40]. The stimulus material was balanced in terms of expressed laughter type (JOY, TAU, TIC) and speaker sex. All stimuli were normalized with respect to mean acoustic energy. Stimulus duration was balanced across laughter types (mean duration ± SD: JOY: 7.56 s±1.59 s; TAU: 7.48 s±1.73 s; TIC: 7.74 s±1.25 s). The resulting stimulus set consisted of 60 laughter sequences (range: 3.2–9.2 s) with 20 stimuli per laughter type. A summary of the acoustic characteristics of the laughter bouts used in the present study is given in Table S1.

### Experimental Design

The fMRI experiment consisted of four runs with 30 trials each within the framework of an event-related design. All stimuli were presented during two different tasks: 1.) explicit processing of social information in the form of a laughter type categorization task (CAT) and 2.) implicit processing of social information in the form of a bout counting task (COU), where participants had to judge how many bouts the laughter sequence consisted of. Participants were instructed to count silently during the bout counting task and not to laugh during the fMRI experiment. A laughter bout was defined as the part of the laughter sequence from the start of a sequence to the first inhaled breath, or the part of the sequence between two inhaled breaths. The fMRI experiment was preceded by a short training session outside the scanner room during which participants practiced both tasks with 12 laughter sequences which were not part of the stimulus set of the main experiment.

During the fMRI experiment, the tasks alternated between runs. The sequence of tasks was balanced across participants. Stimulus presentation was pseudo-randomized within and across runs, balanced for laughter type, number of laughter bouts per sequence, and speaker sex. 120 overall trials were interspersed with 12 null events to decrease the effect of stimulus expectation.

**Figure 1:**
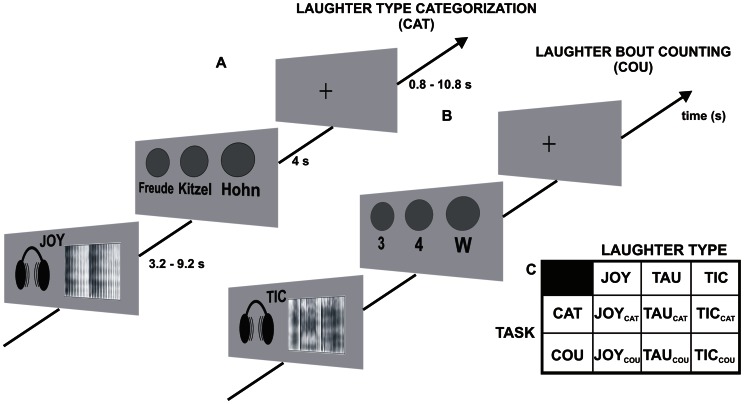
Experimental design. The figure shows two exemplary experimental trials (A, B) and the factorial nature of the design (C). (A) illustrates the laughter type categorization task (CAT) where the participants had to decide which type of laughter they heard: the trial starts with the presentation of a laughter sequence (here: joyful laughter, JOY) followed by a response scale with the three laughter type categories (“Freude” = JOY; “Kitzel” = tickling laughter, TIC; “Hohn” = taunting laughter, TAU) and a variable inter-trial interval. (B) exemplifies the laughter bout counting task (COU) where the participants had to decide of how many laughter bouts the laughter sequence consisted: the laughter sequence (here: TIC) is followed by a response scale with three response categories (“3″, “4″, “W” = any other number of laughter bouts) and the inter-trial interval. Durations on the time axis indicate durations of the stimulus presentation, response window and inter-trial interval. (C) Experimental design: an equal number (n = 20) of JOY, TAU and TIC stimuli are each presented under two task conditions (CAT, COU) leading to total number of 120 trials within an orthogonal factorial design.

Each trial started with the presentation of a laughter sequence which was followed by a horizontal scale with three categories (i.e., joy, taunt, tickle for the laughter type judgment and 3, 4, W (W for neither 3 nor 4) for the laughter bout counting task). Participants had a response window of 4 s to convey their decisions by pressing one of three buttons on a fiber optic system (LumiTouch, Photon Control, Burnaby, Canada) with their right index, middle, or ring finger. The response window was followed by a variable inter-trial interval (range: 0.8 s–10.8 s). This resulted in stimulus onset asynchronies ranging from 14 s to 34 s (null events with a duration of 16 s included). The stimulus onset was jittered relative to the scan onset in steps of 0.5 s ( = ¼ scans). The arrangement of categories on the response scales was fully permuted resulting in six different scales for each task. This was done in order to avoid lateralization effects caused by motor responses or possible laterality effects in the perception of emotionally valenced information. The different scales were balanced across participants. The experimental design is illustrated in Figure 1.

The laughter sequences were presented binaurally via magnetic resonance compatible headphones with piezoelectric signal transmission [41]. Visual cues (fixation cross, classification scale) were back-projected onto a translucent screen (projection size ca. 80×65 cm) placed ca. 2.5 meters from the participants’ head. A mirror system mounted on the head coil allowed participants to view the visual cues.

### Image Acquisition

1200 functional images were recorded for each participant using a 1.5 T whole body scanner (Siemens AVANTO; Siemens, Erlangen, Germany) with an echo-planar imaging (EPI) sequence (repetition time (TR) = 2 s, echo time (TE) = 40 ms, matrix = 64^2^, and flip angle = 90 degrees) covering the whole cerebrum (field of view (FOV) = 192 mm×192 mm, 24 axial slices, 4 mm slice thickness and 1 mm gap, continuous slice acquisition in descending order). Measurements preceding T_1_ equilibrium were excluded by discarding the first 5 EPI images of each run. For offline correction of distortions of the EPI images a static field map (TR = 487 ms, TEs = 5.28 and 10.04 ms) was acquired in every participant. High-resolution T_1_-weighted images were obtained using a magnetization prepared rapid acquisition gradient echo (MPRAGE) sequence (FOV = 256 mm×256 mm, 176 slices, 1-mm slice thickness, no gap, flip angle 15 degrees, TR = 1980 ms, TE = 3.93 ms and matrix size = 256^2^).

### Image Analysis

SPM2 software (Wellcome Department of Imaging Neuroscience, London, UK; http://www.fil.ion.ucl.ac.uk/spm) was used for the analysis of the functional images.

#### Image preprocessing

Prior to statistical analysis of the functional MR images the following preprocessing steps were performed: motion correction, unwarping by use of a static field map, slice time correction to the middle slice (12^th^ slice) and coregistration with the anatomical data. The individual realignment parameters were checked to exclude participants with head motion exceeding 3 mm. However, head motion lay below this critical value for all participants. The MR images were normalized to the Montreal Neurological Institute (MNI) space [42] using a transformation matrix that was calculated on the basis of the structural T1-weighted 3-D data set of each participant and subsequently applied to the functional images (resampled voxel size: 3×3×3 mm^3^). Finally, data were smoothed with a Gaussian filter 10-mm full width half maximum (FWHM).

#### Analytical strategy

As a first step, functional regions of interest (ROI) for the ensuing connectivity analysis were defined based on their differential activation patterns to the degree of complex social information or acoustic complexity imbued in the laughter signal or based on stronger activation during explicit evaluation of social information in laughter via categorical analysis of cerebral responses.

As a second step, dynamic alterations in connectivity due to different degrees of complex social information and acoustic complexity in the laughter signal as well as due to the focusing of attention towards or away from the social information imbedded in the laughter signal were systematically investigated employing a separate psycho-physiological interaction (PPI) analysis taking each of the ROIs as the seed region separately.

#### Categorical analysis of cerebral responses

Each trial was modeled as a separate regressor in the form of a boxcar function with the length of the respective laughter sequence. Thus, each individual model contained 120 event-related regressors. Events were time-locked to stimulus onset. To minimize low-frequency components data were high-pass filtered with a cut-off frequency of 1/128 Hz. The error term was modeled as an autoregressive process with a coefficient of 0.2 [43] and an additional white noise component [44] to account for serial autocorrelations.

Brain regions sensitive to a higher degree of complex social information carried in the laughter signal were identified by contrasting cerebral responses to complex social laughter (CSL = mean of JOY and TAU) types against those to tickling laughter (TIC). The reverse contrast (i.e., TIC vs. CSL) was employed to identify brain regions sensitive to the higher degree of acoustic complexity of tickling laughter. Differential responses to the two CSL types were investigated via the contrasts (JOY> TAU) and (TAU>JOY) in order to detect brain responses specific for the respective CSL type and to detect potential biases in the contrasts of complex social and tickling laughter through only one of the two CSL types. Additionally, areas with stronger cerebral responses during explicit processing of social information in laughter sounds were identified by contrasting cerebral activation under the laughter type categorization (CAT) condition against brain activation under the laughter bout counting condition (COU). Please note that the reverse contrast COU>CAT was not used to define ROIs as it should reveal brain areas involved in counting which the present study expressly was not focused on.

A second-level random effects analysis was performed for the statistical evaluation of group data. Activations are reported at a height threshold of p<0.001, uncorrected, and an extent threshold of k≥25. Corrections for multiple comparisons were performed based on random field theory [45] for the whole brain. For p<0.05, corrected for the family-wise error (FWE) at the cluster level, this corresponds to cluster size thresholds of k≥55 (CSL vs. TIC) and k≥54 (CAT vs. COU).

All regions with differential activation during perception of CSL and TIC or stronger activation during the CAT condition were further tested for interactions between laughter type (CSL/TIC) and task (CAT/COU) on the level of hemodynamic activation in order to identify potential task-specific laughter type effects. To this end, mean parameter estimates were extracted from all differentially activated regions and submitted to a 2×2-factorial analysis of variance (ANOVA) with laughter type (CSL/TIC) and task (CAT/COU) as within-subject factors. All resulting p values were corrected for potential violations of the assumption of sphericity employing the method of Greenhouse and Geisser [46].

In order to investigate potential confounding effects of laughter type-specific effects of task difficulty, an additional parametric analysis modeling task difficulty in a stimulus-wise manner was run. To this end, the mean laughter type categorization and bout counting hit rates from the present experiment were calculated for each stimulus as an estimate of task difficulty for the respective stimulus. Then, contrasts were defined using the stimulus-wise mean hit rates as a parametric regressor. This was done under the assumption that a stimulus with a low hit rate is more difficult to categorize/count than a stimulus with a high hit rate and that there would be a linear relationship between categorization/counting difficulty and the BOLD response. The analysis was performed for each task separately to assess task-specific difficulty effects as well as for both tasks together to assess general effects of task difficulty. Again, second-level random effects analyses were performed with activations reported at a height threshold of p<0.001, uncorrected, and an extent threshold of k≥63 (general task difficulty contrast), k≥51 (CAT difficulty contrast), k≥64 (COU difficulty contrast), corresponding to p<0.05 FWE corrected for multiple comparisons across the whole brain at the cluster level.

#### PPI analyses

As a second step in the analysis, the brain regions exhibiting significant differential responses to CSL and TIC as well as those brain regions with significantly stronger responses during the CAT condition were defined as seed regions for ensuing PPIs. A PPI analysis approach was selected for assessing modulations of connectivity because, in contrast to other approaches for the investigation of cerebral connectivity (e.g., dynamic causal modeling), they allow whole-brain analyses without constraints on the target regions involved in modulations of connectivity with a given seed region. Such an approach appears justified in instances when it is uncertain if all brain regions involved in the cerebral network to be investigated have been reliably identified, which is the case with the cerebral network processing different types of human laughter. For each seed region the enhancement of connectivity during the perception of CSL as opposed to TIC (CSL>TIC), during the perception of TIC as opposed to CSL (TIC>CSL) and during laughter type categorization as opposed to laughter bout counting (CAT>COU) was investigated. Please note that these comparisons between experimental conditions are relative. Thus, a relative enhancement of connectivity under condition A compared to condition B can also be considered as a decrease in connectivity under condition B as compared to condition A. Therefore, in the PPI analysis the contrast CAT<COU was used to investigate decreases in connectivity during laughter type categorization as compared to laughter bout counting. Differential connectivity patterns between the two CSL types were investigated using the contrasts (JOY>TAU) and (TAU>JOY).

In the PPI analyses, the time-course of the BOLD response, based on a sphere with a radius of 3 mm around the individual peak-activation voxel within the respective seed region adjusted for effects of interest was defined as physiological variable. Each experimental event (i.e., laughter sequence) was defined as a separate psychological input variable. These were then contrasted to achieve the following contrasts between different laughter types and tasks (CSL>TIC, TIC>CSL, JOY>TAU, TAU>JOY or CAT>COU). The PPI was calculated as the product of the deconvolved activation time course [38] and the vector of the psychological variables. Through the deconvolution of the BOLD response with the hemodynamic response function it is possible to assess psychophysiological interactions at the neuronal level. This is useful in experimental settings with low frequency stimulation like event-related designs.

The physiological and psychological variables and the psychophysiological interaction term were then entered as three separate regressors into a single SPM model. Please note that the algorithm implemented in SPM2 orthogonalizes the regressors within the model by default, rendering the PPI term independent of the physiological and psychological variables. This may considerably reduce the sensitivity of the PPI analysis in cases where these variables are correlated, but it also effectively prevents circular results.

Again, a second-level random effects analysis was performed for the statistical evaluation of PPI group data. Changes in connectivity were assessed using two approaches:

#### ROI-based PPI analyses

In an approach similar to von Kriegstein and Giraud [47], each of the PPI seed regions was also defined as target region in a ROI-based approach. This set of ROIs which were differentially modulated by the experimental factors (laughter type, task) was complemented by a set of additional target ROIs which were activated under all experimental conditions during laughter perception. These ROIs were defined by a six-fold conjunction analysis with a conjunction null hypothesis [48] across the main effects of all experimental conditions (JOY_CAT_ ∩ TAU_CAT_ ∩ TIC_CAT_ ∩ JOY_COU_ ∩ TAU_COU_ ∩ TIC_COU_) excluding regions differentially activated by laughter type or task. As the conjunction analysis was based on the main effects of all experimental conditions, a strict height threshold of p<0.0001, uncorrected, was employed to allow spatial differentiation of commonly activated regions. Together with the extent threshold of k>15 voxels, this corresponds to p<0.05, FWE corrected for multiple comparisons across the whole brain at the cluster level. Changes in connectivity are reported at a statistical threshold of p<0.05, corrected for multiple comparisons across the respective target ROI (small volume correction, [49]) with a height threshold of p<0.001, uncorrected and cluster size of k≥5. For a strict control of the alpha error, resulting p values were then additionally Bonferroni-corrected for the number of investigated connections between ROIs (15 seed ROIs×20 target ROIs each = 300 connections).

#### Whole-brain PPI analyses

A set of whole-brain PPI analyses (CSL>TIC, TIC>CSL, JOY>TAU, TAU>JOY and CAT>COU) were performed for each seed region. Here, statistical significance was assessed using an uncorrected height threshold of p<0.001 at the voxel level and a FWE correction (p<0.05) for multiple comparisons across the whole brain at the cluster level. Exact cluster size thresholds are given in tables S6, S7, and S8. Additionally, p values were Bonferroni-corrected for the number of seed regions (15) to prevent alpha error inflation.

Additionally, ROI analyses centered on the bilateral amygdalae (as defined by the AAL toolbox, [50]) were performed with heightened sensitivity (height threshold p<0.01 and extent threshold k≥3) for all contrasts of interest (see above) both on the level of hemodynamic activation as well as for the connectivity analyses. Here, the right and left amygdalae were defined as additional target ROIs in the PPI analyses of each of the functionally defined seed regions. Resulting p values were small volume corrected for the right or left amygdala, respectively, and Bonferroni corrected for the number of amygdalae (i.e., 2).

## Results

### Behavioral Data

The laughter type categorization task (CAT) yielded the following performance rates (mean hit rates with SEM in parentheses): JOY: 76.7% (3.5%), TAU: 80.6% (3.5%), TIC: 63.3% (4.2%). In the bout counting condition (COU) the subsequent counting performance rates were determined for the three laughter types: JOY: 89.4% (0.9%), TAU: 96.7% (1.0%), TIC: 74.2% (1.5%). One-sample t-tests indicated that the participants were able to perform well above chance level (33%) under both task conditions and for all laughter types with all t(17)≥7.2 and all p<0.001. Taunting and joyful laughter were categorized with comparable accuracy (t(17) = 0.9, p = 0.385) while both complex social laughter types were categorized with higher accuracy than tickling laughter (JOY vs. TIC: t(17) = 2.4, p = 0.030; TAU vs. TIC: t(17) = 4.1, p = 0.001). Counting hit rates were higher for both taunting and joyful laughter than for tickling laughter (JOY vs. TIC: t(17) = 8.2, p<0.001; TAU vs. TIC: t(17) = 13.4, p<0.001) and for taunting laughter higher than for joyful laughter (t(17) = 5.6, p<0.001). The bout counting task yielded higher hit rates than the laughter type categorization task: 86.8% (0.7%) (COU), 73.5% (2.5%) (CAT) (t(17) = 4.9, p<0.001). Reaction times, however, did not differ between the two tasks: 812 ms (52 ms) (COU), 808 ms (73 ms) (CAT) (t(17) = 0.1, p = 0.909).

### Neuroimaging Data

Categorical analysis of cerebral responses – Definition of ROIs for the connectivity analysis.

**Figure 2:**
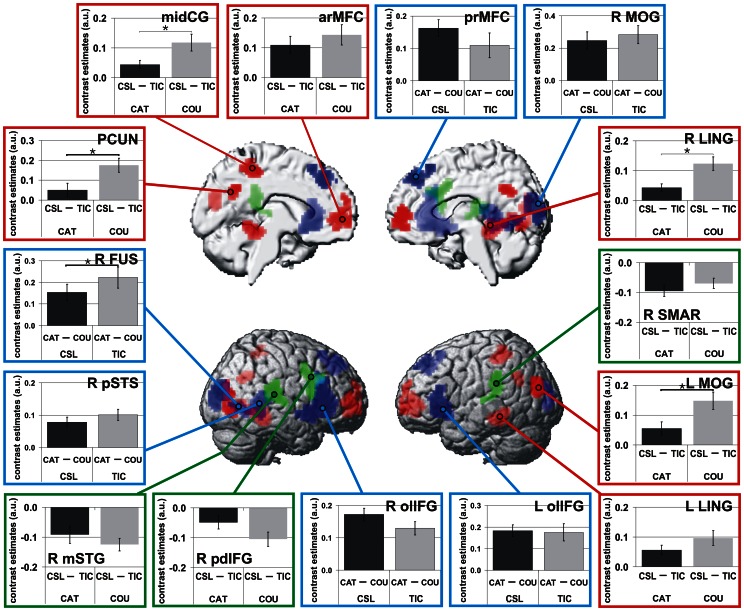
Laughter type- and task-dependent cerebral responses defining ROIs for connectivity analyses. Increased responses to complex social laughter types (CSL>TIC, red), to tickling laughter (TIC>CSL, green) and during explicit processing of social information of laughter (CAT>COU, blue) (p<0.001, uncorrected, cluster size k≥55 (CSL vs. TIC) and k≥54 (CAT vs. COU), corresponding to p<0.05, FWE corrected at cluster level). Panels depict mean contrast estimates extracted from activated regions. Please note that displayed effects are relative contrasts and do not correspond to general hemodynamic activations or deactivations. Asterisks mark significant interactions (p<0.05) between laughter type (CSL/TIC) and task (CAT/COU). Activations are rendered on an MNI standard brain.

Perception of CSL, associated with more complex social information, led to significantly stronger activation as compared to TIC within several midline structures, namely the bilateral anterior rostral medial frontal cortex (arMFC), the left middle cingulate cortex (midCG) and the bilateral precuneus (PCUN) as well as within the bilateral lingual/fusiform gyri (R/L LING) and the left middle occipital gyrus (L MOG) extending into the angular and middle temporal gyri (Table 1; Figure 2 red). Acoustically more complex TIC elicited significantly stronger brain responses than CSL within the posterior dorsal part of the right IFG extending into the middle frontal gyrus (R pdIFG) as well as within the middle part of the right superior temporal gyrus (R mSTG) and the left supramarginal gyrus extending into the superior temporal gyrus (L SMAR; Table 1; Fig. 1 green). A task-related increase of activation during the CAT condition (CAT>COU) associated with explicit processing of social information in the laughter sounds could be observed within the bilateral orbitolateral parts of the inferior frontal gyrus (R/L olIFG), the right posterior superior temporal sulcus (pSTS), the right fusiform gyrus extending into the calcarine gyrus (R FUS), the right middle occipital gyrus extending into the right superior occipital and right calcarine gyri (R MOG) and the bilateral posterior rostral mediofrontal cortex (prMFC; Table 1; Figure 2 blue).

**Table 1:** Differential hemodynamic activation following the perception of complex social laughter types (CSL) and tickling laughter (TIC) and stronger hemodynamic activation following explicit evaluation of laughter type.

	x	y	z	Z-score (peak voxel)	Cluster size (voxel)
*LAUGHTER TYPE EFFECTS*					
*CSL>TIC*					
R lingual gyrus/R fusiform gyrus/R middle occipital gyrus/R inferior frontal gyrus/R calcarine gyrus/R middle temporal gyrus/R inferior temporal gyrus (ROI: R LING)	30	−45	−6	4.48	254*
L lingual gyrus/L parahippocampal gyrus/L fusiform gyrus/L hippocampus (ROI: L LING)	−24	−45	−6	4.42	106*
L middle occipital gyrus/L angular gyrus/L middle temporal gyrus (ROI: L MOG)	−42	−81	21	4.30	57*
R+L superior frontal gyrus, medial/R+L medial orbital gyrus/L superior frontal gyrus/R+Lanterior cingulum (ROI: arMFC)	9	54	6	4.25	230*
L middle cingulum/L Precuneus/L paracentral lobule (ROI: midCG)	−12	−39	51	4.24	87*
R postcentral gyrus/R superior parietal gyrus	21	−39	63	3.97	27
L middle temporal gyrus/L inferior temporal gyrus	−54	−6	−18	3.87	28
L+R precuneus/L cuneus/R posterior cingulum (ROI: PCUN)	−6	−57	33	3.60	81*
R angular gyrus/R middle occipital gyrus	48	−69	33	3.57	29
*TIC>CSL*					
R inferior frontal gyrus p. triangularis and p. opercularis/R middle frontal gyrus/R precentralgyrus (ROI: R pdIFG)	36	15	30	4.48	141*
R superior temporal gyrus/R supramarginal gyrus (ROI: R mSTG)	63	−30	18	4.41	117*
L supramarginal gyrus/L superior temporal gyrus/L Rolandic operculum (ROI: L SMAR)	−60	−36	33	4.06	97*
R thalamus	6	−18	3	3.90	32
*TASK EFFECT*					
*CAT>COU*					
R inferior frontal gyrus p. triangularis, p. opercularis and p. orbitalis/insula/superior temporalpole/Rolandic operculum (ROI: R olIFG)	51	27	9	5.25	426*
L inferior frontal gyrus p. orbitalis and p. triangularis/insula (ROI: L olIFG)	−42	24	−6	5.03	260*
R superior temporal gyrus/R middle temporal gyrus (ROI: R pSTS)	45	−45	3	4.54	105*
R middle occipital gyrus/R superior occipital gyrus/R calcarine gyrus/R cuneus (ROI: R MOG)	27	−87	18	4.44	216*
R+L medial superior frontal gyrus/R+L supplementary motor area (ROI: prMFC)	3	39	48	4.42	154*
R fusiform gyrus/R lingual gyrus/R calcarine gyrus (ROI: R FUS)	30	−60	−3	3.74	63*
L middle frontal gyrus/inferior frontal gyrus p. triangularis and p. opercularis	−42	21	33	3.54	34
R+L cerebellum	12	−81	−18	3.44	34

Activations thresholded at p<0.001, uncorrected with a cluster size k>25 voxels. Coordinates refer to the MNI system.

* p<0.05, FWE corrected for multiple comparisons across the whole brain at the cluster level.

The comparison of brain responses following perception of joyful and taunting laughter sounds was performed to detect potential biases in the contrasts of CSL and TIC through one of the two CSL types. This comparison did not yield any significant differences (all p>0.05, FWE corrected at the cluster level with a height-threshold of p<0.001, uncorrected).

Within several posterior brain regions, significant interactions between laughter type and task indicated differential responses to CSL and TIC dependant upon the attentional focus of the task: R LING, L MOG, PCUN and midCG exhibited a significantly stronger increase of activity for CSL as compared to TIC during the COU condition (all F(1,17)≥5.3, p≤0.03; Figure 2). In R FUS, on the other hand, a significantly stronger increase in cerebral responses during CAT was observed for TIC as compared to CSL (F(1,17) = 5.6, p = 0.03; Figure 2).

The amygdala ROI analysis did not yield any significant differential activation for laughter types or task (see Table S2).

**Figure 3:**
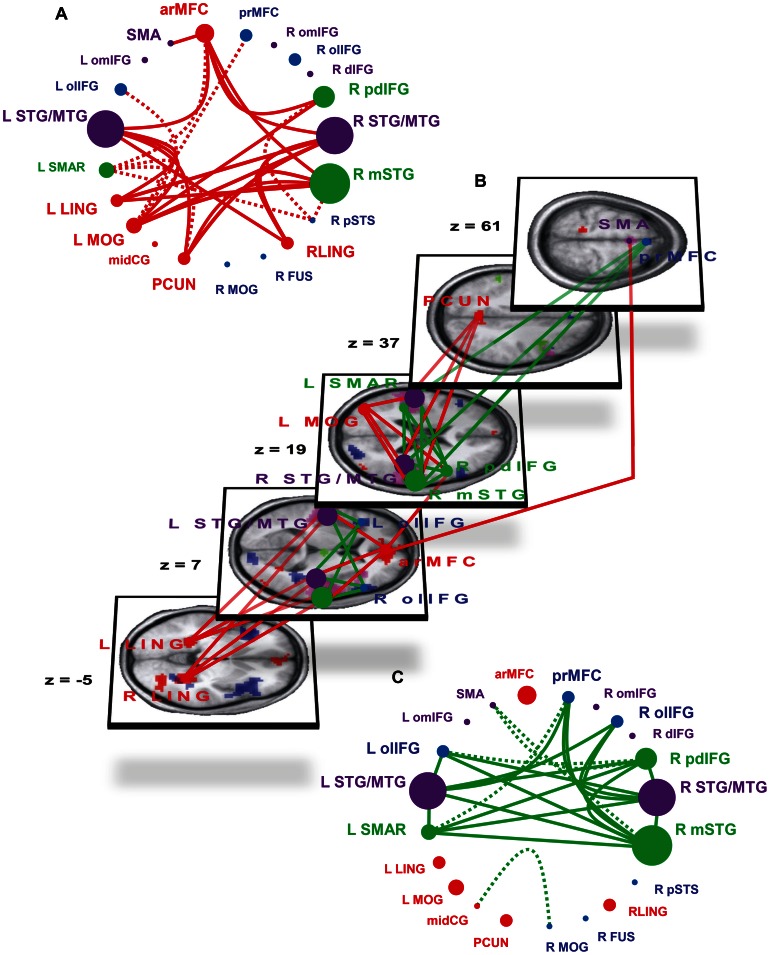
Connectivity modulations within the laughter perception network through complex social laughter types and tickling laughter. Brain regions with significantly increased responses to CSL (CSL>TIC; red areas/dots), to tickling laughter (TIC>CSL; green areas/dots) and during explicit processing of social information of laughter (CAT>COU; blue areas/dots) as well as regions with equal activation under all experimental conditions (mauve areas/dots) are shown in schematic form (A, C) and superimposed on a three dimensional rendering of five transversal slices of the subjects’ mean anatomic image (B). Increased connectivity during perception of CSL (CSL>TIC; red lines; A and B), and during TIC perception (TIC>CSL; green lines; B and C). Continuous lines: modulations of connectivity which survive correction for multiple comparisons within the target ROI and additional Bonferroni-correction for the number of investigated connections (300). Broken lines: modulations which survive correction for multiple comparisons within the target ROI but not Bonferroni-correction and for which the activated portion of the target ROI is part of a significant target cluster of the whole-brain analysis. Z coordinates refer to the MNI-system. The size of the dots symbolizing the separate ROIs is scaled according to the number of Bonferroni-corrected significant modulations of connectivity of the respective ROI.

**Figure 4:**
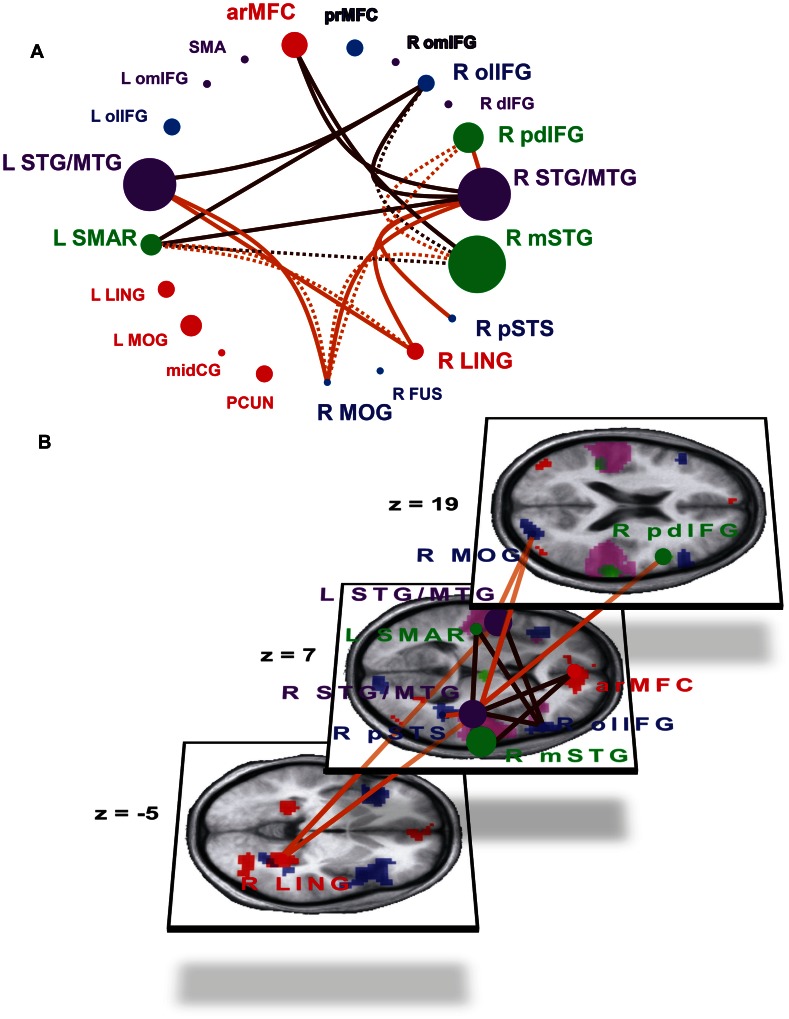
Differences in connectivity within the laughter perception network during perception of joyful (JOY) and taunting (TAU) laughter. Brain regions with significantly increased responses to CSL (CSL>TIC; red areas/dots), to TIC (TIC>CSL; green areas/dots) and during explicit processing of social information of laughter (CAT>COU; blue areas/dots) as well as regions with equal activation under all experimental conditions (mauve areas/dots) are shown in schematic form (A) and superimposed on a three dimensional rendering of three transversal slices of the subjects’ mean anatomic image (B). Increased connectivity during perception of joyful laughter (JOY>TAU; orange-brown lines; A, B), and during taunting laughter perception (TAU>JOY; dark brown lines; A, B). Continuous lines: modulations of connectivity which survive correction for multiple comparisons within the target ROI and additional Bonferroni-correction for the number of investigated connections (300). Broken lines: modulations which survive correction for multiple comparisons within the target ROI but not Bonferroni-correction; additionally, the activated portion of the target ROI is part of a significant target cluster of the whole-brain analysis. Z coordinates refer to the MNI-system.

The conjunction analysis (JOY_CAT_ ∩ TAU_CAT_ ∩ TIC_CAT_ ∩ JOY_COU_ ∩ TAU_COU_ ∩ TIC_COU_) identified the following six brain regions commonly and comparably activated by all experimental conditions: large parts of the bilateral primary auditory and auditory association cortex (R and L STG/MTG), bilateral areas in the orbitomedial part of the IFG bordering on the anterior part of the insula (R and L omIFG), an area in the dorsal part of the right IFG (R dIFG) and a region in the supplementary motor area (SMA; see Table 2, Figure 3 B and Figure 4 B).

**Table 2:** Regions with common hemodynamic activation for complex social laughter types (CSL) and reflex-like tickling laughter (TIC) during explicit evaluation of laughter type and laughter bout counting which did not show any differential hemodynamic activation between different laughter types or task conditions.

	x	y	z	Z-score (peak voxel)	Cluster size (voxel)
*HAP_CAT_ ∩ TAU_CAT_ ∩ TIC_CAT_ ∩ HAP_COU_ ∩ TAU_COU_ ∩ TIC_COU_*					
R superior temporal gyrus/R Rolandic operculum/R Heschl’s gyrus/R supramarginalgyrus/R middle temporal gyrus/R postcentral gyrus/R insula	51	−15	6	6.35	704
L superior temporal gyrus/L Rolandic operculum/L supramarginal gyrus/L postcentralgyrus/L Heschl’s gyrus	−54	−15	12	6.04	657
R gyrus frontalis inferior p. opercularis/R middle frontal gyrus	48	15	33	4.63	16
R gyrus frontalis inferior p. triangularis/R insula	33	27	6	4.48	41
R+L supplementary motor area	3	6	63	4.41	42
L gyrus frontalis inferior p. triangularis/L insula	−33	24	12	4.30	22

Activations thresholded at p<0.0001, uncorrected with a cluster size k>15 voxels, corresponding to p<0.05, FWE corrected for multiple comparisons across the whole brain at the cluster level. Coordinates refer to the MNI system.

No significant impact of task-specific as well as general difficulty of task performance on cerebral responses could be observed using parametric whole-brain analyses with stimulus-wise estimates of task difficulty (all p>0.05, FWE corrected at the cluster level with a height-threshold of p<0.001, uncorrected).

#### Psycho-Physiological Interaction (PPI) analyses - ROI-based analyses

Complex social information-containing CSL significantly enhanced connectivity between R mSTG, R STG/MTG and L STG/MTG, on the one hand, and almost all brain regions with stronger responses to CSL (arMFC, R and L LING, L MOG, PCUN), on the other, with the sole exception of midCG. Moreover, CSL enhanced connectivity between R pdIFG and L MOG as well as arMFC and between arMFC and SMA (Figure 3 A and B, continuous red lines; Table S3). Acoustically more complex TIC enhanced connectivity among all three regions sensitive to this laughter type (R pdIFG, R mSTG, L SMAR) and between each of these and R as well as L STG/MTG. Moreover, TIC enhanced connectivity between R mSTG, R STG/MTG and L STG/MTG and three regions with stronger responses to explicit evaluation of social information in laughter (R and L olIFG, prMFC; Figure 3 B and C, continuous green lines; Table S3).

While the two exemplars of CSL employed in the present study, i.e., JOY and TAU, did not elicit any differential hemodynamic activation, these complex social laughter types modulated connectivity differently in the laughter perception network: JOY elicited an increase in connectivity between R and L STG/MTG and R LING and R MOG. Additional increases in connectivity through JOY were observed between R STG/MTG and R pSTS and R pdIFG (Figure 4, orange-brown lines, Table S4). TAU, on the other hand, was accompanied by increases in connectivity between bilateral STG/MTG and L SMAR and R olIFG. Additional TAU-associated increases in connectivity were observed between R STG/MTG and arMFC and L SMAR as well as between R mSTG and arMFC.

No significant task-related modulations of connectivity were observed, however (Table S5).

#### Whole-brain analyses

This set of analyses was used to investigate modulations of connectivity outside the network of regions with experimentally modulated hemodynamic activation and to double check the ROI analyses at the whole-brain level.

While in the ROI-analyses 38 (20 CSL>TIC; 18 TIC>CSL) of 300 investigated connections were found to be differentially modulated by CSL and TIC, the whole-brain analyses yielded a total of 47 significant target clusters where CSL or TIC modulated the connectivity with one of the 15 seed regions (see Table S6). A close comparison between the ROI- and whole-brain analyses indicated that virtually all modulations of connectivity through different laughter types corresponded to significant clusters in the whole-brain analyses. Furthermore, 13 significant clusters of the whole-brain analyses exhibited a considerable overlap with regions of interest from the ROI approach where the respective modulation of connectivity had been rejected as insignificant due to Bonferroni-correction (Figure 3 A, C, broken lines; Table S3, colored cell frames).

Finally, it was found that CSL and TIC modulated connectivity between the PPI seed regions and six brain regions which were spatially distinct from the study’s ROIs. CSL increased connectivity between R pdIFG, R mSTG and L SMAR and three strongly overlapping regions in the right temporo-occipito-parietal junction (Table S6). Furthermore, CSL enhanced connectivity between the following regions: R pSTS and a posterior dorsal part of the left IFG extending into middle frontal gyrus and precentral gyrus – a left hemispheric homologue of the R pdIFG-ROI; L LING and left caudate nucleus and thalamus. TIC, in contrast, enhanced connectivity between R MOG and a region in the left middle frontal gyrus extending into the superior frontal gyrus.

For the comparison between JOY and TAU, the whole-brain analyses (see Table S7) gave no evidence of connectivity modulations within regions spatially distinct from the ROIs. In fact, on top of confirming every significant modulation of the ROI analyses, seven additional significant clusters from the whole-brain analyses exhibited a significant overlap with R mSTG, R pdIFG, L SMAR, R MOG and R LING. These overlapping findings indicate significant modulations of connectivity between these regions through JOY and TAU which had been rejected in the Bonferroni-correction of the ROI analyses (Table S7, Figure 4 A, broken lines; Table S4, colored cell frames).

Concordant with the ROI analyses, no significant task-related modulations of connectivity were found (Table S8).

Parallel to the negative results on the level of hemodynamic activation, no significant modulations of connectivity between any of the 15 seed regions and the amygdala through any of the experimental contrasts could be observed in the additional ROI-analysis (Table S9).

## Discussion

Using a whole-brain approach in the present series of analyses, we were able to considerably extend our previously published findings [7] on the neural correlates underlying the processing of different types of human laughter both on the level of hemodynamic activation and connectivity.

### Laughter Type-dependent and Task-dependent Hemodynamic Responses

Compared to our previous report [7], the present whole-brain analysis of hemodynamic activation demonstrated additional differential responses in occipital and parietal brain regions. A tickling laughter-sensitive area was found at the left temporo-parietal junction (L SMAR) positioned more posterior than its right hemispheric counterpart (R mSTG).

Stronger responses to complex social laughter types were found in the precuneus/posterior cingulum (PCUN) and middle cingulum/precuneus (midCG), areas which have repeatedly been described as parts of the mentalizing or theory of mind network [51], [52]. These can be interpreted parallel to those responses in the arMFC as resulting from the greater capacity of these laughter types to trigger mentalizing processes. Interestingly, the response differences between complex social laughter types and tickling laughter in PCUN and midCG are significantly stronger under the task condition when attention is diverted from the socio-relational information of the laughter signal. This indicates that complex social laughter types may automatically trigger such mentalizing processes. A reason for this, beyond the greater amount of potential socio-relational implications of joyful and taunting laughter, could be that complex social laughter types occurs more often and in a much greater variety of social situations where they are processed implicitly but still with the need for swift and correct interpretation. This contextual factor may have lead to an even greater sensitivity of the mentalizing system to complex social laughter types in contrast to tickling laughter, as tickling laughter typically occurs in a narrower spectrum of situations and incurs a lower need for mentalizing. The explicit evaluation of social information in the laughter signal during the categorization task, on the other hand, can be expected to trigger mentalizing processes regardless of the perceived laughter type, thus reducing the observed activation differences during the categorization condition.

A plausible interpretation for the finding of stronger responses to complex social laughter types in the visual association cortex is that visual imagery may be elicited in connection with or as part of the mentalizing processes triggered by complex social laughter types. With the loci of activations within the occipito-temporal junction and the medial temporal cortex, areas well known to harbor face processing areas [53], [54], facial imagery would appear as the most likely form of imagery involved [55], [56]. With respect to laughter perception, Meyer and colleagues [14] reported a similar effect with stronger responses in the fusiform gyrus when comparing perception of laughter to non-vocal and non-biological sounds which they also discussed in relation to facial imagery.

Two of the three complex social laughter type-sensitive areas in the visual association cortex of the left occipito-temporal junction (L MOG) and bilateral lingual/fusiform gyri (R and L LING) exhibited an activation pattern parallel to the one observed in PCUN and midCG with a non-significant interaction in L LING. Here, the parallel activation pattern of posterior mentalizing areas and visual association areas supports the notion of a connection between these activations, possibly with facial imagery supporting the decoding of social intentions.

Finally, the detection of two task-sensitive areas in the visual association cortex of the right hemisphere suggests that visual imagery is also involved in the explicit evaluation of social information in laughter, formalized here as laughter type classification.

However, the spatial distinction of areas sensitive to complex social laughter types and those sensitive to explicit evaluation of social information in the laughter signal clearly shows that the surmised mentalizing processes triggered by complex social laughter types and the explicit social evaluation of laughter are not equivalent even though they may share certain components, as suggested by the observed interactions between laughter type and task.

The lack of observed modulations of hemodynamic responses in the amygdala stands in contrast to the findings of Sander and colleagues [11]–[13] but is in line with the results of Meyer and colleagues [14]. There is a methodological difference between the present and previous studies which might explain this discrepancy: similar to the study by Meyer and colleagues, the stimuli used in the present study were very short compared to those used by Sander and colleagues. Meyer and colleagues argued that insufficient emotional induction may be the reason for the lack of amygdala activation.

### Connectivity

#### Increased connectivity for complex social laughter types

In contrast to the somewhat generic increase in connectivity between regions sensitive to complex social laughter types and the auditory cortex, a small number of connectivity increases outside the auditory cortex stand out distinctly. We propose that these increases in connectivity between anterior mediofrontal cortex (arMFC), left occipito-temporal junction (L MOG) and right posterior superior temporal sulcus (R pSTS), on the one hand, and right dorsolateral prefrontal cortex (R pdIFG), on the other, may offer a perspective on the neurofunctional processes linking mentalizing (arMFC; [8], [57]–[60], visual imagery (L MOG), explicit evaluation of social information in laughter (R pSTS) and auditory attention [23], [61] and working memory processes [24], [25], [62], [63] of auditory information, all linked to the dorsolateral prefrontal cortex. Further, the increases in connectivity between left occipito-temporal junction and left ventrolateral prefrontal cortex (L olIFG) and posterior rostral mediofrontal cortex (prMFC) may reflect the association of visual imagery (L MOG) with social evaluation (olIFG) and attention and action monitoring (prMFC) during perception of complex social laughter types.

The synopsis from ROI-based analyses and whole-brain analyses suggests that apparent hemispheric differences in the connectivity patterns of tickling-laughter sensitive auditory regions (R mSTG and L SMAR; Figure 3 A, broken red lines) may be the result of strict statistical alpha-error control in the ROI-approach with concomitant beta-error inflation and not a relevant laterality effect. The inclusion of brain regions commonly activated by human laughter in the analysis demonstrate that the increases in connectivity are in no way specific for tickling laughter-sensitive areas in the auditory cortex but rather encompass large parts of the auditory cortex generally activated during laughter perception.

The most prominent findings of the whole-brain connectivity analyses outside the study’s ROIs were highly consistent increases in connectivity between a region at the right temporo-occipito-parietal junction and the tickling laughter-sensitive areas in bilateral auditory association cortex (R mSTG and L SMAR) and right dorsolateral prefrontal cortex (R pdIFG). Judging from inspection of contrast maxima and pattern of modulated connections, this region could be a right hemisphere homologue of L MOG. Although lacking the increased responses during perception of complex social laughter types, it could potentially be involved in enhanced visual imagery during processing of complex social laughter types.

#### Increased connectivity for tickling laughter

Tickling laughter perception led to enhanced connectivity among different regions in the bilateral auditory association cortex (R mSTG, L SMAR, R and L STG/MTG), on the one hand, and between the auditory association cortex and the right dorsolateral prefrontal cortex (pdIFG), the bilateral ventrolateral prefrontal cortex (olIFG) and the posterior rostral mediofrontal cortex (prMFC), on the other. For R mSTG and R pdIFG an additional increase in connectivity with the supplementary motor area (SMA) was observed.

The emergence of this second functional subnetwork centered on the bilateral auditory association cortex in the context of tickling laughter perception may reflect the influence of the increased processing effort that the characteristics of tickling laughter (i.e., higher acoustic complexity and greater information transfer rate, [9] (see also Table S1)) impose on the laughter perception network. The fact that virtually all involved temporal and frontal regions are subject to enhanced connectivity with the auditory association cortex of the R mSTG might depict how the higher acoustic information transfer rate of tickling laughter automatically leads to a more intensive acoustic analysis. This analysis appears to be processed within a neural network entailing brain regions related to the extraction of supra-segmental acoustic information (mSTG; [20], [64]), to auditory attention and working memory (pdIFG) and to evaluation processes (olIFG). In spite of the fact that the ventrolateral prefrontal cortex (olIFG) does not count among the regions with stronger responses to tickling laughter than to complex social laughter types, the observed enhancement in connectivity here could be due to a higher acoustic information load during the evaluation process associated with tickling laughter.

Importantly, the occurrence of enhanced connectivity between the right middle superior temporal cortex (R mSTG) and the right dorsolateral prefrontal cortex (R pdIFG) during perception of tickling laughter corroborates previous observations of Leitman and colleagues demonstrating that coupling between these areas increases with decreasing stimulus saliency [35]. This increase in connectivity might reflect sensory tuning and increased attentional processes when stimuli are more ambiguous.

The enhancement in connectivity between the auditory association cortex and the prMFC could similarly be interpreted as the result of more difficult response selection given the lower stimulus saliency of tickling laughter. Increased connectivity between right middle superior temporal cortex (R mSTG) as well as right dorsolateral prefrontal cortex (R pdIFG) and the supplementary motor area could be seen as corroboration of a model discussed by Gervais and Wilson [6]. This model predicts that the specific perception of unintentional or so-called Duchenne laughter would involve the laughter motor program supposedly represented in the supplementary motor area.

The most consistent feature of the observed connectivity patterns is mainly that the connections between regions in the auditory cortex and other brain regions are modulated by different laughter types. This, in itself, is not surprising given the acoustic nature of auditory laughter perception. However, this general pattern highlights the potential significance of connectivity modulations outside the auditory cortex for the neural processing of different laughter types: here, the right dorsolateral prefrontal cortex (R pdIFG) stands out particularly in terms of “connectedness” in both functional subnetworks. Its connectivity pattern highlights this structure as a potentially pivotal network node storing meaningful sound patterns and linking them to visual imagery, thus facilitating inference on social intentions.

Keeping in mind that of the different brain regions implicated in the networks modulated by complex social laughter types, on the one hand, and tickling laughter, on the other hand, only a few display stronger responses to the respective laughter types, it becomes obvious that the classical categorical analysis of BOLD responses only portrays the “tip of the iceberg” of laughter processing. Changes in connectivity have until now remained “below the waterline”. The changes in functional coupling between brain regions subserving different aspects of laughter processing induced by one type of laughter, and even within partly overlapping neural subnetworks induced by different laughter types, offer a novel perspective on the neural substrates of laughter perception.

#### Differential connectivity patterns for joyful and taunting laughter

It is a surprising finding of the present study that differences between cerebral responses to joyful and taunting laughter could not be observed at the level of hemodynamic contrasts but were clearly present at the level of connectivity modulations. The lack of differential hemodynamic responses to two laughter types communicating distinct socio-relational information with considerable differences in valence, social dominance and arousal in this first fMRI-experiment encompassing several types of laughter is in itself not very surprising in light of the literature on nonverbal vocal expressions of different emotions using speech melody. Studies over the past two decades have demonstrated differential activation patterns for the presence or absence of nonverbally communicated emotional information but consistently failed to find reliable, specific hemodynamic activation patterns for separate emotions using categorical univariate approaches [27], [64].

Recently, however, it was demonstrated that different types of emotional speech melody can be discriminated using a multivariate pattern analysis [65], [66], showing that information aiding the discrimination of the neural signatures of different vocal expressions of emotions can be acquired from widespread multi-voxel patterns across the brain rather than from focal activations. With respect to the present study, there is a considerable overlap between those brain regions found to be informative in the discrimination of different types of emotional speech melody by Kotz and colleagues [66] and those regions in the present study with specific connectivity patterns discriminating between joyful and taunting laughter including right posterior and anterior STG/MTG, left posterior MTG, right frontal operculum and more dorsal and posterior parts of the right IFG and an anterior mediofrontal region.

Keeping in mind the common denominator of the two studies, i.e., the use of cerebral responses from spatially distinct and distal brain areas to discriminate between different categories of vocal expressions, both studies suggest that focal activation differences may not be sufficient for discrimination of cerebral responses to specific types of vocal expressions in neuroimaging studies. Rather, they provide consistent evidence that information from spatially distal areas needs to be combined to achieve this goal. Secondly, the overlap in informative regions between the two studies might implicate that a similar set of brain structures may be involved in discriminating between types of emotional speech melody and types of complex social laughter types. With respect to the lack of significant modulations of connectivity of the amygdala through different laughter types, the same potential causes have to be discussed as for the observed lack of differences in hemodynamic activation (see above).

#### Task-dependent modulations of connectivity

For task-directed shifts of attention to or away from explicit evaluation of social information of the laughter stimuli, no significant effect on connectivity between the different parts of the laughter perception network could be observed.

This lack of connectivity modulations by a shift in attentional focus to the explicit evaluation of social information supports the concept that, considered from the perspective of connectivity, the perception of laughter may trigger processes of social evaluation irrespective of task requirements. This idea also fits with the finding that the assumed neural correlates of mentalizing processes induced by complex social laughter types are independent of task-dependent shifts of attention [7].

### Limitations and Perspectives

In terms of directionality or causality, the interpretations of the observed connectivity patterns in the present study have to be treated as tentative due to the fact that PPI analyses neither enable definite inferences on directionality of connectivity nor on the underlying structural connections.

Although no influence of the difficulty of task performance on hemodynamic responses could be observed, behavioral response patterns did indicate differences in task difficulty between tickling laughter and complex social laughter types as well as between tasks. Thus, higher task difficulty for tickling laughter and the laughter type categorization task could potentially influence the functional coupling of brain regions and the interaction between laughter type and task. In order to improve the disambiguation of the effects of laughter type and attentional focus on functional connectivity patterns from those of differential task difficulty, further studies with more strictly difficulty-matched stimulus material would be desirable. Additionally, individual stimulus-wise response times could be used as a control measure.

As the stimulus-material of the present study consisted of laughter portrayals produced by professional actors, it may be questioned if these portrayals are equivalent to spontaneously produced laughs. Although some authors state that vocal portrayals of emotion may represent prototypical and more intense expressions and overemphasize acoustical characteristics, the majority of authors in the literature on vocal communication of emotion assume the equivalence of portrayals to natural vocalizations [67], [68]. Moreover, with regard to laughter, it was demonstrated that it is very difficult to distinguish between “faked” and spontaneous laughter based on the acoustic structure [69], which is well in line with the finding that the acoustic properties of portrayed laughter are mostly equivalent to those of spontaneous laughter [9]. Nevertheless, the question if the cerebral correlates of perception of spontaneous and portrayed laughter differ remains to be answered in further studies.

Keeping in mind that for a meaningful analysis of connectivity modulations in a network of brain regions associated with a certain cognitive function a comprehensive detection and definition of these functional ROIs is necessary, recent methodological advances in data analysis may prove very useful for future research. Multivariate analysis of spatial activation patterns associated with different experimental conditions has been demonstrated to be useful for the definition of functional ROIs for connectivity analyses [70]. As it appears to be more sensitive than classical univariate analysis approaches, in future studies this technique may therefore afford a more complete definition of the set of brain regions in which the activation is modulated as a function of task conditions or stimulus types.

Finally, for further studies on auditory laughter perception the employment of localizer experiments for face-sensitive brain regions could be very helpful to gain further insight into the implications of differential hemodynamic activations through different laughter types in the visual associative cortex.

### Conclusion

Complex socio-relational information and acoustic complexity carried in different types of human laughter modulate connectivity in two distinguishable but partially overlapping parts of the laughter perception network irrespective of task instructions.

Connectivity changes presumably related to the higher acoustic complexity of tickling laughter occurred between dorsolateral as well as ventrolateral parts of the IFG, prMFC and the auditory association cortex. They may reflect more intensive acoustic analysis associated with similarly increased demands on auditory attention, working memory, evaluation and response selection processes.

In contrast, connectivity modulations through the higher degree of socio-relational information of complex social laughter types affected connections between auditory association cortices, the right dorsolateral IFG and brain areas linked to mentalizing and visual imagery. These may depict the interconnection of the automatic analysis of informative acoustic features, attention direction to certain aspects of the laughter signal and the retention of this information in working memory during evaluation processes supported by visual imagery as the basis for social cognition processes. The right dorsolateral IFG in this scheme acts as a network node potentially linking the functions of auditory and visual associative sensory cortices with those of mentalizing-associated arMFC.

Finally, despite the lack of focal differential hemodynamic activation patterns for joyful and taunting laughter, significantly different connectivity patterns were found for these complex social laughter types. This once more highlights the value of the combined analysis of cerebral responses from spatially distinct brain regions, here instantiated in the form of connectivity analyses, in the research on the neural underpinnings of social perception.

## Supporting Information

Table S1 **Acoustic characterization of laughter types.**
(DOC)

Table S2 **ROI analysis of the bilateral amygdalae.** Differential hemodynamic activation following the perception of complex social laughter types (CSL) and reflex-like tickling laughter (TIC) and stronger hemodynamic activation following explicit evaluation of social information in laughter (CAT>COU).
(DOC)

Table S3 **Effects of complex social (CSL) and of tickling (TIC) laughter on connectivity within the laughter perception network as assessed by psycho-physiological interaction analyses (PPI).**
(DOC)

Table S4 **Effects of explicit versus implicit evaluation of social information in the laughter signal (CAT>COU; COU>CAT) on connectivity within the laughter perception network as assessed by psycho-physiological interaction analyses (PPI).**
(DOC)

Table S5 **Effects of joyful and taunting laughter on connectivity within the laughter perception network as assessed by psycho-physiological interaction analyses (PPI).**
(DOC)

Table S6 **Whole-brain analyses.** Relative changes in cerebral functional connectivity (PPI) associated with complex social laughter types (CSL) and reflex-like tickling laughter (TIC).
(DOC)

Table S7 **Whole-brain analyses.** Relative changes in cerebral functional connectivity (PPI) associated with the perception of different types of complex social laughter (joyful - JOY, taunting - TAU).
(DOC)

Table S8 **Whole-brain analyses.** Relative changes in cerebral functional connectivity (PPI) associated with explicit evaluation of laughter type (CAT) as compared to laughter bout counting (COU).
(DOC)

Table S9 **ROI analysis of the bilateral amygdalae.** Relative changes in cerebral functional connectivity (PPI) following the perception of complex social laughter types (CSL), reflex-like tickling laughter (TIC), different complex social laughter types (JOY, TAU) and explicit versus implicit evaluation of laughter type (CAT,COU).
(DOC)

Sound S1 **Exemplar of joyful laughter.**
(WAV)

Sound S2 **Exemplar of tickling laughter.**
(WAV)

Sound S3 **Exemplar of taunting laughter.**
(WAV)
