# Supplementary material for: Different Types of Laughter Modulate Connectivity within Distinct Parts of the Laughter Perception Network
Source: PLoS One. 2013 May 8;8(5):e63441. doi: 10.1371/journal.pone.0063441 (PMC3648477; doi:10.1371/journal.pone.0063441)
Supplement: Table S1 — Acoustic characterization of laughter types. (DOC) [file pone.0063441.s001.doc]

**Table S1:** Acoustic characterization of laughter types

| ***LAUGHTER TYPES*** | **JOY** | **TAU** | **TIC** |  |
| --- | --- | --- | --- | --- |
| ***ACOUSTIC PARAMETERS*** | mean (S.D.) | mean (S.D.) | mean (S.D.) | comparison of laughter types |
| f0 (Hz) | 339 | 240 | 461 | CSL < TIC** |
|  | (182) | (93) | (229) |  |
| f1 (Hz) | 751 | 941 | 751 | CSL = TIC |
|  | (122) | (181) | (201) |  |
| f2 (Hz) | 1548 | 1626 | 1734 | CSL < TIC** |
|  | (134) | (162) | (227) |  |
| amplitude variability (S.D. of db) | 0.085 | 0.109 | 0.091 | CSL = TIC |
|  | (0.010) | (0.023) | (0.016) |  |
| harmonics-to-noise-ratio | 8.5 | 6.9 | 9.6 | CSL < TIC* |
|  | (3.5) | (2.5) | (3.7) |  |
| phrase duration (s) | 2.55 | 2.06 | 1.69 | CSL > TIC* |
|  | (1.22) | (0.65) | (0.75) |  |
| inter phrase duration (s) | 0.68 | 0.49 | 0.37 | CSL > TIC** |
|  | (0.36) | (0.15) | (0.13) |  |
| syllable duration (s) | 0.090 | 0.102 | 0.081 | CSL > TIC*** |
|  | (0.011) | (0.016) | (0.012) |  |
| inter syllable duration (s) | 0.208 | 0.213 | 0.185 | CSL > TIC* |
|  | (0.018) | (0.017) | (0.012) |  |
| laugh rate (syllables/s) | 4.13 | 4.20 | 4.72 | CSL < TIC*** |
|  | (0.65) | (0.41) | (0.54) |  |

Note: f0 = fundamental frequency; f1 and f2 = first and second formant; phrase = laughter bout; syllable = single laughter phoneme (e.g. “ha”); CSL = mean(JOY, TAU); asterisks mark results of T-tests for independent samples: * = p < 0.05, ** = p < 0.01, *** = p < 0.001
