# Supplementary material for: Different Types of Laughter Modulate Connectivity within Distinct Parts of the Laughter Perception Network
Source: PLoS One. 2013 May 8;8(5):e63441. doi: 10.1371/journal.pone.0063441 (PMC3648477; doi:10.1371/journal.pone.0063441)
Supplement: Table S2 — ROI analysis of the bilateral amygdalae. Differential hemodynamic activation following the perception of complex social laughter types (CSL) and reflex-like tickling laughter (TIC) and stronger hemodynamic activation following explicit evaluation of social information in laughter (CAT>COU). (DOC) [file pone.0063441.s002.doc]

**Table S2:** ROI analysis of the bilateral amygdalae: Differential hemodynamic activation following the perception of complex social laughter types (CSL) and reflex-like tickling laughter (TIC) and stronger hemodynamic activation following explicit evaluation of social information in laughter (CAT>COU).

|  | **x** | **y** | **z** | **Z-score (peak voxel)** | **Cluster size (voxel)** |
| --- | --- | --- | --- | --- | --- |
| ***STIMULUS EFFECTS*** |  |  |  |  |  |
| ***CSL > TIC -*** No cluster above threshold |  |  |  |  |  |
| ***TIC > CSL*** |  |  |  |  |  |
| R Amygdala | - | - | - | - | - |
| L Amygdala | -18 | -3 | -12 | 2.70 | 7 |
| ***TASK EFFECT*** |  |  |  |  |  |
| ***CAT > COU*** |  |  |  |  |  |
| R Amygdala | 24 | -9 | -12 | 2.50 | 4 |
| L Amygdala | - | - | - | - | - |

Activations thresholded at p < 0.01, uncorrected with a cluster size k ≥ 3 voxels. Coordinates refer to the MNI system.
