# Supplementary material for: Different Types of Laughter Modulate Connectivity within Distinct Parts of the Laughter Perception Network
Source: PLoS One. 2013 May 8;8(5):e63441. doi: 10.1371/journal.pone.0063441 (PMC3648477; doi:10.1371/journal.pone.0063441)
Supplement: Table S3 — Effects of complex social (CSL) and of tickling (TIC) laughter on connectivity within the laughter perception network as assessed by psycho-physiological interaction analyses (PPI). (DOC) [file pone.0063441.s003.doc]

**Table S3:** Effects of complex social (CSL) and of tickling (TIC) laughter on connectivity within the laughter perception network as assessed by psycho-physiological interaction analyses (PPI):

| **TARGET/SEED** | **R pdIFG** | **R mSTG** | **L SMAR** | **R LING** | **L LING** | **L MOG** | **arMFC** | **midCG** | **PCUN** | **R olIFG** | **L olIFG** | **R pSTS** | **R MOG** | **prMFC** | **R FUS** |  |
| --- | --- | --- | --- | --- | --- | --- | --- | --- | --- | --- | --- | --- | --- | --- | --- | --- |
| **R pdIFG** |  | n.s. | n.s. | n.s. | n.s. | n.s. | **p=0.036** |  | n.s. | n.s. | n.s. | n.s. |  | n.s. | n.s. | **R pdIFG** |
|  |  | Z = 3.56 | Z = 3.74 | Z = 3.69 | Z = 3.98 | Z = 3.84 | **Z = 3.78** |  | Z = 3.71 | Z = 3.56 | Z = 3.95 | Z = 4.31 |  | Z = 4.12 | Z = 3.41 |  |
| **R mSTG** | **p=0.004** |  | **p=0.013** | **p=0.015** | **p=0.002** | **p=0.015** | **p=0.003** |  | **p=0.008** | **p=0.029** | **p=0.014** | n.s. | n.s. | **p=0.007** | n.s. | **R mSTG** |
|  | **Z = 5.19** |  | **Z = 4.51** | **Z = 4.05** | **Z = 4.12** | **Z = 3.94** | **Z = 4.51** |  | **Z = 4.3** | **Z = 4.22** | **Z = 4.18** | Z = 3.69 | Z = 3.73 | **Z = 4.09** | Z = 3.29 |  |
| **L SMAR** | **p=0.045** | n.s. |  | n.s. | n.s. | n.s. | n.s. |  | n.s. | n.s. | n.s. | n.s. | n.s. | n.s. |  | **L SMAR** |
|  | **Z = 4.18** | Z = 4.06 |  | Z = 3.66 | Z = 4.04 | Z = 4.26 | Z = 4.66 |  | Z = 4.58 | Z = 3.91 | Z = 3.46 | Z = 3.72 | Z = 3.22 | Z = 3.4 |  |  |
| **R LING** | n.s. | n.s. | n.s. |  |  |  |  |  |  |  | n.s. |  |  | n.s. |  | **R LING** |
|  | Z = 3.69 | Z = 4.65 | Z = 3.85 |  |  |  |  |  |  |  | Z = 3.71 |  |  | Z = 3.81 |  |  |
| **L LING** |  | n.s. |  |  |  |  |  |  |  |  |  |  |  |  |  | **L LING** |
|  |  | Z = 4.15 |  |  |  |  |  |  |  |  |  |  |  |  |  |  |
| **L MOG** | **p=0.034** | n.s. | n.s. |  |  |  |  |  |  | n.s. | n.s. |  |  | n.s. |  | **L MOG** |
|  | **Z = 4.29** | Z = 4.57 | Z = 4.75 |  |  |  |  |  |  | Z = 3.54 | Z = 4.75 |  |  | Z = 3.87 |  |  |
| **arMFC** |  | **p=0.009** | n.s. |  |  |  |  |  |  |  | n.s. |  |  | n.s. |  | **arMFC** |
|  |  | **Z = 4.4** | Z = 4.02 |  |  |  |  |  |  |  | Z = 3.29 |  |  | Z = 3.5 |  |  |
| **midCG** |  |  |  |  |  |  |  |  |  |  |  |  | n.s. |  |  | **midCG** |
|  |  |  |  |  |  |  |  |  |  |  |  |  | Z = 3.65 |  |  |  |
| **PCUN** |  | **p=0.031** | n.s. |  |  |  |  |  |  |  |  |  |  | n.s. |  | **PCUN** |
|  |  | **Z = 4.08** | Z = 3.92 |  |  |  |  |  |  |  |  |  |  | Z = 3.44 |  |  |
| **R olIFG** | n.s. |  | n.s. |  | n.s. | n.s. | n.s. |  | n.s. |  | n.s. | n.s. |  | n.s. |  | **R olIFG** |
|  | Z = 3.86 |  | Z = 3.46 |  | Z = 3.54 | Z = 3.29 | Z = 3.82 |  | Z = 3.92 |  | Z = 3.46 | Z = 3.64 |  | Z = 3.7 |  |  |
| **L olIFG** |  |  |  |  |  |  |  |  |  |  |  | n.s. |  |  |  | **L olIFG** |
|  |  |  |  |  |  |  |  |  |  |  |  | Z = 3.6 |  |  |  |  |
| **R pSTS** |  |  | n.s. |  |  |  |  |  | n.s. | n.s. | n.s. |  |  |  |  | **R pSTS** |
|  |  |  | Z = 3.50 |  |  |  |  |  | Z = 3.89 | Z = 3.69 | Z = 3.47 |  |  |  |  |  |
| **R MOG** |  |  |  |  |  |  |  |  |  |  |  |  |  |  |  | **R MOG** |
|  |  |  |  |  |  |  |  |  |  |  |  |  |  |  |  |  |
| **prMFC** |  |  |  |  |  |  |  |  |  |  |  |  |  |  |  | **prMFC** |
|  |  |  |  |  |  |  |  |  |  |  |  |  |  |  |  |  |
| **R FUS** |  |  |  |  |  |  |  |  |  |  |  |  |  |  |  | **R FUS** |
|  |  |  |  |  |  |  |  |  |  |  |  |  |  |  |  |  |
| **R STG/MTG** | **p<0.001** | **p<0.001** | **p<0.001** | **p<0.001** | **p<0.001** | **p<0.001** | **p<0.001** | n.s. | **p<0.001** | **p<0.001** | **p<0.001** | n.s. | n.s. | **p<0.001** | n.s. | **R STG/MTG** |
|  | **Z = 5.22** | **Z = 4.64** | **Z = 5.09** | **Z = 4.05** | **Z = 4.38** | **Z = 4.26** | **Z = 4.66** | Z = 3.56 | **Z = 5.05** | **Z = 4.90** | **Z = 3.95** | Z = 3.71 | Z = 3.79 | **Z = 4.11** | Z = 3.4 |  |
| **L STG/MTG** | **p<0.001** | **p<0.001** | **p<0.001** | **p=0.001** | **p<0.001** | **p<0.001** | **p<0.001** | n.s. | **p<0.001** | **p<0.001** | **p=0.005** | n.s. | n.s. | **p=0.001** | n.s. | **L STG/MTG** |
|  | **Z = 4.46** | **Z = 4.29** | **Z = 4.21** | **Z = 3.91** | **Z = 4.68** | **Z = 4.40** | **Z = 4.70** | Z = 4.61 | **Z = 4.57** | **Z = 4.62** | **Z = 3.86** | Z = 3.91 | Z = 3.43 | **Z = 4.19** | Z = 3.59 |  |
| **R omIFG** | n.s. |  |  | n.s. |  | n.s. | n.s. |  | n.s. | n.s. | n.s. |  |  |  |  | **R omIFG** |
|  | Z = 4.12 |  |  | Z = 3.17 |  | Z = 3.38 | Z = 3.72 |  | Z = 3.22 | Z = 4.16 | Z = 3.39 |  |  |  |  |  |
| **L omIFG** |  |  |  |  |  |  |  |  |  | n.s. |  |  |  |  |  | **L omIFG** |
|  |  |  |  |  |  |  |  |  |  | Z = 3.28 |  |  |  |  |  |  |
| **R dIFG** |  |  | n.s. | n.s. |  |  | n.s. |  | n.s. |  | n.s. | n.s. |  |  |  | **R dIFG** |
|  |  |  | Z = 3.76 | Z = 3.45 |  |  | Z = 3.74 |  | Z = 3.86 |  | Z = 3.59 | Z = 3.68 |  |  |  |  |
| **SMA** | n.s. | n.s. | n.s. | n.s. | n.s. |  | **p=0.044** |  | n.s. |  | n.s. |  |  |  |  | **SMA** |
|  | Z = 3.6 | Z = 3.77 | Z = 3.59 | Z = 3.2 | Z = 3.76 |  | **Z = 4.61** |  | Z = 3.41 |  | Z = 3.68 |  |  |  |  |  |

CSL>TIC = red fields, TIC>CSL = green fields. Z values indicate the statistical maximum of the connectivity increase in the respective ROI. P values are corrected for multiple comparisons within the respective ROI and additionally Bonferroni-corrected for the number of investigated connections (300). Darker colors mark results which survive Bonferroni-correction. Results in light shade colors do not survive Bonferroni-correction and are listed for the sake of completeness. Colored cell frames appear where a non-significant connectivity increase for the respective target ROI was part of a significant cluster from the whole brain PPI analyses. Colored ROI names indicate the nature of significant hemodynamic effects within the respective ROI: stimulus driven (CSL > TIC = red, TIC > CSL = green), task driven (CAT > COU = blue) or common activation under all experimental conditions (mauve).
