# Supplementary material for: Different Types of Laughter Modulate Connectivity within Distinct Parts of the Laughter Perception Network
Source: PLoS One. 2013 May 8;8(5):e63441. doi: 10.1371/journal.pone.0063441 (PMC3648477; doi:10.1371/journal.pone.0063441)
Supplement: Table S4 — Effects of explicit versus implicit evaluation of social information in the laughter signal (CAT>COU; COU>CAT) on connectivity within the laughter perception network as assessed by psycho-physiological interaction analyses (PPI). (DOC) [file pone.0063441.s004.doc]

**Table S4:** Effects of explicit versus implicit evaluation of social information in the laughter signal (CAT > COU; COU > CAT) on connectivity within the laughter perception network as assessed by psycho-physiological interaction analyses (PPI):

| **TARGET/SEED** | **R pdIFG** | **R mSTG** | **L SMAR** | **R LING** | **L LING** | **L MOG** | **arMFC** | **midCG** | **PCUN** | **R olIFG** | **L olIFG** | **R pSTS** | **R MOG** | **prMFC** | **R FUS** |  |
| --- | --- | --- | --- | --- | --- | --- | --- | --- | --- | --- | --- | --- | --- | --- | --- | --- |
| **R pdIFG** |  |  |  |  |  |  |  | n.s. |  |  |  |  |  |  |  | **R pdIFG** |
|  |  |  |  |  |  |  |  | Z = 3.47 |  |  |  |  |  |  |  |  |
| **R mSTG** |  |  |  |  |  |  |  |  |  |  |  |  |  |  |  | **R mSTG** |
|  |  |  |  |  |  |  |  |  |  |  |  |  |  |  |  |  |
| **L SMAR** |  |  |  |  |  |  |  |  |  |  |  |  |  |  |  | **L SMAR** |
|  |  |  |  |  |  |  |  |  |  |  |  |  |  |  |  |  |
| **R LING** |  |  |  |  |  |  |  |  |  |  |  |  |  |  |  | **R LING** |
|  |  |  |  |  |  |  |  |  |  |  |  |  |  |  |  |  |
| **L LING** |  |  |  |  |  |  |  |  |  |  |  |  |  |  |  | **L LING** |
|  |  |  |  |  |  |  |  |  |  |  |  |  |  |  |  |  |
| **L MOG** |  |  |  |  |  |  |  |  |  |  |  |  |  |  |  | **L MOG** |
|  |  |  |  |  |  |  |  |  |  |  |  |  |  |  |  |  |
| **arMFC** |  |  |  |  |  |  |  |  |  |  |  |  |  |  |  | **arMFC** |
|  |  |  |  |  |  |  |  |  |  |  |  |  |  |  |  |  |
| **midCG** |  |  |  |  |  |  |  |  |  |  |  |  |  |  |  | **midCG** |
|  |  |  |  |  |  |  |  |  |  |  |  |  |  |  |  |  |
| **PCUN** |  |  |  |  |  |  |  |  |  |  |  |  |  |  |  | **PCUN** |
|  |  |  |  |  |  |  |  |  |  |  |  |  |  |  |  |  |
| **R olIFG** |  |  |  |  |  |  |  |  |  |  |  |  |  |  |  | **R olIFG** |
|  |  |  |  |  |  |  |  |  |  |  |  |  |  |  |  |  |
| **L olIFG** |  |  |  |  |  |  |  |  |  |  |  |  |  |  |  | **L olIFG** |
|  |  |  |  |  |  |  |  |  |  |  |  |  |  |  |  |  |
| **R pSTS** |  |  |  |  |  |  |  |  |  |  |  |  |  |  |  | **R pSTS** |
|  |  |  |  |  |  |  |  |  |  |  |  |  |  |  |  |  |
| **R MOG** |  |  |  |  |  |  |  |  |  |  |  |  |  |  |  | **R MOG** |
|  |  |  |  |  |  |  |  |  |  |  |  |  |  |  |  |  |
| **prMFC** |  |  |  |  |  |  |  |  |  |  |  |  |  |  |  | **prMFC** |
|  |  |  |  |  |  |  |  |  |  |  |  |  |  |  |  |  |
| **R FUS** |  |  |  |  |  |  |  |  |  |  |  |  |  |  |  | **R FUS** |
|  |  |  |  |  |  |  |  |  |  |  |  |  |  |  |  |  |
| **R STG/MTG** |  |  |  |  |  |  |  |  |  |  |  |  |  |  |  | **R STG/MTG** |
|  |  |  |  |  |  |  |  |  |  |  |  |  |  |  |  |  |
| **L STG/MTG** |  |  |  |  |  |  |  |  |  |  |  |  |  |  |  | **L STG/MTG** |
|  |  |  |  |  |  |  |  |  |  |  |  |  |  |  |  |  |
| **R omIFG** |  |  |  |  |  |  |  |  |  |  |  |  |  |  |  | **R omIFG** |
|  |  |  |  |  |  |  |  |  |  |  |  |  |  |  |  |  |
| **L omIFG** |  |  |  |  |  |  |  |  | n.s. |  |  |  |  |  |  | **L omIFG** |
|  |  |  |  |  |  |  |  |  | Z = 3.77 |  |  |  |  |  |  |  |
| **R dIFG** |  |  |  |  |  |  |  |  |  |  |  |  |  |  |  | **R dIFG** |
|  |  |  |  |  |  |  |  |  |  |  |  |  |  |  |  |  |
| **SMA** |  |  |  |  |  |  |  |  |  |  |  |  |  |  |  | **SMA** |
|  |  |  |  |  |  |  |  |  |  |  |  |  |  |  |  |  |

CAT > COU = blue fields. Z values indicate the statistical maximum of the connectivity increase in the respective ROI. P values are corrected for multiple comparisons within the respective ROI and additionally Bonferroni-corrected for the number of investigated connections (300). Results in light shade colors do not survive Bonferroni-correction and are listed for the sake of completeness. Colored ROI names indicate the nature of significant hemodynamic effects within the respective ROI: stimulus driven (CSL > TIC = red, TIC > CSL = green), task driven (CAT > COU = blue) or common activation under all experimental conditions (mauve).
