# Supplementary material for: Different Types of Laughter Modulate Connectivity within Distinct Parts of the Laughter Perception Network
Source: PLoS One. 2013 May 8;8(5):e63441. doi: 10.1371/journal.pone.0063441 (PMC3648477; doi:10.1371/journal.pone.0063441)
Supplement: Table S5 — Effects of joyful and taunting laughter on connectivity within the laughter perception network as assessed by psycho-physiological interaction analyses (PPI). (DOC) [file pone.0063441.s005.doc]

**Table S5:** Effects of joyful and taunting laughter on connectivity within the laughter perception network as assessed by psycho-physiological interaction analyses (PPI):

| **TARGET/SEED** | **R pdIFG** | **R mSTG** | **L SMAR** | **R LING** | **L LING** | **L MOG** | **arMFC** | **midCG** | **PCUN** | **R olIFG** | **L olIFG** | **R pSTS** | **R MOG** | **prMFC** | **R FUS** |  |
| --- | --- | --- | --- | --- | --- | --- | --- | --- | --- | --- | --- | --- | --- | --- | --- | --- |
| **R pdIFG** |  |  |  |  |  |  |  |  |  |  |  |  | n.s. |  |  | **R pdIFG** |
|  |  |  |  |  |  |  |  |  |  |  |  |  | Z = 3.92 |  |  |  |
| **R mSTG** | n.s. |  | n.s. |  |  |  | **p=0,02** |  |  | n.s. | n.s. |  | n.s. |  |  | **R mSTG** |
|  | Z = 4.08 |  | Z = 4.59 |  |  |  | **Z = 3.68** |  |  | Z = 3.91 | Z = 3.48 |  | Z = 3.67 |  |  |  |
| **L SMAR** |  |  |  | n.s. |  |  |  |  |  | **p=0,001** |  |  | n.s. |  |  | **L SMAR** |
|  |  |  |  | Z = 3.88 |  |  |  |  |  | **Z = 4.10** |  |  | Z = 3.50 |  |  |  |
| **R LING** |  |  |  |  |  |  |  |  |  |  | n.s. |  |  |  |  | **R LING** |
|  |  |  |  |  |  |  |  |  |  |  | Z = 3.59 |  |  |  |  |  |
| **L LING** |  | n.s. |  |  |  |  |  |  |  |  |  |  |  |  |  | **L LING** |
|  |  | Z = 4.15 |  |  |  |  |  |  |  |  |  |  |  |  |  |  |
| **L MOG** |  |  |  |  |  |  |  |  |  |  |  |  |  |  |  | **L MOG** |
|  |  |  |  |  |  |  |  |  |  |  |  |  |  |  |  |  |
| **arMFC** |  |  |  |  |  |  |  |  |  |  |  |  |  |  |  | **arMFC** |
|  |  |  |  |  |  |  |  |  |  |  |  |  |  |  |  |  |
| **midCG** |  |  |  |  |  |  |  |  |  |  |  |  |  |  |  | **midCG** |
|  |  |  |  |  |  |  |  |  |  |  |  |  |  |  |  |  |
| **PCUN** |  |  |  |  |  |  |  |  |  |  |  |  |  |  |  | **PCUN** |
|  |  |  |  |  |  |  |  |  |  |  |  |  |  |  |  |  |
| **R olIFG** |  |  |  | n.s. |  |  |  |  |  |  |  | n.s. | n.s. |  |  | **R olIFG** |
|  |  |  |  | Z = 3.66 |  |  |  |  |  |  |  | Z = 3.34 | Z = 3.66 |  |  |  |
| **L olIFG** |  |  |  |  |  |  |  |  |  |  |  |  | n.s. |  |  | **L olIFG** |
|  |  |  |  |  |  |  |  |  |  |  |  |  | Z = 3.58 |  |  |  |
| **R pSTS** |  |  |  |  |  |  |  |  |  |  |  |  |  |  |  | **R pSTS** |
|  |  |  |  |  |  |  |  |  |  |  |  |  |  |  |  |  |
| **R MOG** |  |  |  |  |  |  |  |  |  |  |  |  |  |  |  | **R MOG** |
|  |  |  |  |  |  |  |  |  |  |  |  |  |  |  |  |  |
| **prMFC** |  |  |  |  |  |  |  |  |  |  |  |  |  |  |  | **prMFC** |
|  |  |  |  |  |  |  |  |  |  |  |  |  |  |  |  |  |
| **R FUS** |  |  |  |  |  |  |  |  |  |  |  |  |  |  |  | **R FUS** |
|  |  |  |  |  |  |  |  |  |  |  |  |  |  |  |  |  |
| **R STG/MTG** | **p=0.007** |  | **p=0.001** | **p=0.002** |  |  | **p=0.045** | n.s. |  | **p=0.020** | n.s. | **p=0.019** | **p<0.001** |  |  | **R STG/MTG** |
|  | **Z = 4.2** |  | **Z = 4.47** | **Z = 4.13** |  |  | **Z = 3.79** | Z = 3.45 |  | **Z = 3.88** | Z = 3.39 | **Z = 3.86** | **Z = 4.07** |  |  |  |
| **L STG/MTG** |  |  | n.s. | **p<0.001** |  |  | n.s. |  |  | **p=0.025** |  | n.s. | **p=0.02** |  |  | **L STG/MTG** |
|  |  |  | Z = 3.99 | **Z = 3.92** |  |  | Z = 3.8 |  |  | **Z = 4.07** |  | Z = 3.73 | **Z = 4.18** |  |  |  |
| **R omIFG** |  |  |  |  |  |  |  |  |  |  |  |  | n.s. |  |  | **R omIFG** |
|  |  |  |  |  |  |  |  |  |  |  |  |  | Z = 3.88 |  |  |  |
| **L omIFG** |  |  |  |  |  |  |  |  |  |  |  |  | n.s. |  |  | **L omIFG** |
|  |  |  |  |  |  |  |  |  |  |  |  |  | Z = 3.44 |  |  |  |
| **R dIFG** |  |  |  |  |  |  |  |  |  |  |  |  | n.s. |  | n.s. | **R dIFG** |
|  |  |  |  |  |  |  |  |  |  |  |  |  | Z = 3.55 |  | Z = 3.53 |  |
| **SMA** | n.s. |  |  | n.s. |  |  |  |  |  |  |  | n.s. | n.s. |  | n.s. | **SMA** |
|  | Z = 3.52 |  |  | Z = 4.03 |  |  |  |  |  |  |  | Z = 3.30 | Z = 3.87 |  | Z = 3.38 |  |

JOY > TAU = orange-brown fields, TAU > JOY = dark brown fields. Z values indicate the statistical maximum of the connectivity increase in the respective ROI. P values are corrected for multiple comparisons within the respective ROI and additionally Bonferroni-corrected for the number of investigated connections (300). Darker colors mark results which survive Bonferroni-correction. Results in light shade colors (JOY > TAU = light orange, TAU > JOY = yellow) do not survive Bonferroni-correction and are listed for the sake of completeness. Colored cell frames appear where a non-significant connectivity increase for the respective target ROI was part of significant cluster from the whole brain PPI analyses. Colored ROI names indicate the nature of significant hemodynamic effects within the respective ROI: stimulus driven (CSL > TIC = red, TIC > CSL = green), task driven (CAT > COU = blue) or common activation under all experimental conditions (mauve).
