# Supplementary material for: Different Types of Laughter Modulate Connectivity within Distinct Parts of the Laughter Perception Network
Source: PLoS One. 2013 May 8;8(5):e63441. doi: 10.1371/journal.pone.0063441 (PMC3648477; doi:10.1371/journal.pone.0063441)
Supplement: Table S6 — Whole-brain analyses. Relative changes in cerebral functional connectivity (PPI) associated with complex social laughter types (CSL) and reflex-like tickling laughter (TIC). (DOC) [file pone.0063441.s006.doc]

**Table S6:** Whole-brain analyses: Relative changes in cerebral functional connectivity (PPI) associated with complex social laughter types (CSL) and reflex-like tickling laughter (TIC) as investigated with a separate whole-brain analysis for each seed ROI (see Tab. 1):

|  | **x** | **y** | **Z** | **Z-score (peak voxel)** | **Cluster size (voxel)** |
| --- | --- | --- | --- | --- | --- |
| ***CSL > TIC*** |  |  |  |  |  |
| ***SEED: R LING*** |  |  |  |  |  |
| R superior temporal gyrus/ R Rolandic operculum/ R supramarginal gyrus/ R Heschl’s gyrus ***[R mSTG, R STG/MTG]*** | 63 | -39 | 18 | 4.06 | 518* |
| R caudate nucleus | 12 | 12 | 3 | 3.92 | 81 |
| L postcentral gyrus/ L superior temporal gyrus/ L Rolandic operculum/ L supramarginal gyrus/ L Heschl’s gyrus ***[L STG/MTG]*** | -54 | -18 | 18 | 3.91 | 242* |
| R inferior frontal gyrus p. opercularis and p. triangularis/ R precentral gyrus | 45 | 12 | 27 | 3.69 | 62 |
| ***SEED: L LING*** |  |  |  |  |  |
| L superior temporal gyrus/ L supramarginal gyrus/ L Rolandic operculum/ L postcentral gyrus/ L Heschl’s gyrus/ L middle temporal gyrus ***[L STG/MTG]*** | -60 | -39 | 15 | 4.68 | 485* |
| R Heschl’s gyrus/ R superior temporal gyrus/ R Rolandic operculum/ R supramarginal gyrus/ R temporal pole/ R insula ***[R mSTG, R STG/MTG]*** | 51 | -9 | 3 | 4.38 | 754* |
| L inferior frontal gyrus p. opercularis and p. triangularis/ L precentral gyrus/ L middle frontal gyrus | -36 | 9 | 30 | 4.23 | 56 |
| L supplementary motor area/ L superior frontal gyrus/ L paracentral lobule | -9 | 6 | 60 | 3.97 | 66 |
| L+R thalamus/ L caudate nucleus | -3 | 0 | 12 | 3.88 | 165* |
| ***SEED: L MOG*** |  |  |  |  | [>195] |
| L superior temporal gyrus/ L Rolandic operculum/ L supramarginal gyrus/ L postcentral gyrus/ L Heschl’s gyrus ***[L STG/MTG]*** | -60 | -18 | 9 | 4.40 | 364* |
| R superior temporal gyrus/ R Rolandic operculum/ R Heschl’s gyrus ***[R mSTG, R STG/MTG]*** | 51 | -30 | 12 | 4.26 | 428* |
| ***SEED: arMFC*** |  |  |  |  | [>150] |
| R supramarginal gyrus/ R superior temporal gyrus/ R rolandic operculum/ R middle temporal gyrus/ R Heschl’s gyrus/ R temporal pole ***[R mSTG, R STG/MTG]*** | 51 | -45 | 24 | 5.13 | 865* |
| L superior temporal gyrus/ L supramarginal gyrus/ L Rolandic operculum/ L Heschl’s gyrus / L postcentral gyrus ***[L STG/MTG]*** | -51 | -18 | 9 | 4.70 | 604* |
| R inferior frontal gyrus p. triangularis and p. opercularis/ R insula/ R precentral gyrus/ R middle frontal gyrus ***[R pdIFG]*** | 39 | 24 | 15 | 4.52 | 231* |
| R+L supplementary motor area/ R superior frontal gyrus ***[SMA]*** | 9 | 15 | 57 | 4.31 | 167* |
| L precentral gyrus/ L inferior frontal gyrus p. opercularis/ L middle frontal gyrus | -39 | 3 | 33 | 4.09 | 62 |
|  | **x** | **y** | **Z** | **Z-score (peak voxel)** | **Cluster size (voxel)** |
| ***CSL > TIC (continued)*** |  |  |  |  |  |
| ***SEED: arMFC*** |  |  |  |  | [>195] |
| R caudate nucleus/ R thalamus/ R pallidum | 12 | 3 | 6 | 4.06 | 68 |
| L caudate nucleus/ L putamen | -15 | 9 | 9 | 4.03 | 56 |
| ***SEED: midCG*** |  |  |  |  |  |
| No cluster above threshold |  |  |  |  |  |
| ***SEED: PCUN*** |  |  |  |  |  |
| R rolandic operculum/ R superior temporal gyrus/ R insula/ R supramarginal gyrus/ R Heschl’s gyrus/ R temporal pole ***[R mSTG, R STG/MTG]*** | 66 | -12 | 12 | 5.05 | 634* |
| L superior temporal gyrus/ L supramarginal gyrus/ L postcentral gyrus / L Rolandic operculum/ L middle temporal gyrus ***[L STG/MTG]*** | -48 | -33 | 18 | 4.58 | 490* |
| R inferior frontal gyrus p. opercularis and p. triangularis/ R precentral gyrus/ R middle frontal gyrus | 48 | 12 | 36 | 3.83 | 87 |
| ***SEED: R pdIFG*** |  |  |  |  |  |
| L middle occipital gyrus/ L angular gyrus /L middle temporal gyrus ***[L MOG]*** | -42 | -75 | 27 | 4.29 | 196* |
| R middle occipital gyrus/ R middle temporal gyrus / R angular gyrus | 39 | -66 | 12 | 3.87 | 191* |
| ***SEED: R mSTG*** |  |  |  |  |  |
| L angular gyrus/ L middle occipital gyrus/ L middle temporal gyrus/ L inferior parietal gyrus ***[L MOG]*** | -42 | -72 | 42 | 4.77 | 540* |
| L+R superior frontal gyrus, medial/ L+R anterior cingulum/ R+L superior frontal gyrus, medial orbital/ L+R superior frontal gyrus ***[arMFC]*** | 6 | 42 | 3 | 4.59 | 583* |
| R middle temporal gyrus/ R temporal pole | -45 | 3 | -27 | 4.55 | 66 |
| R middle occipital gyrus/ R angular gyrus/ R middle temporal gyrus | 48 | -75 | 30 | 4.54 | 230* |
| L+R Precuneus/ L+R middle and posterior cingulum ***[PCUN]*** | -6 | -54 | 36 | 4.08 | 252* |
| L middle frontal gyrus/ L superior frontal gyrus | -30 | 27 | 51 | 3.99 | 70 |
| ***SEED: L SMAR*** |  |  |  |  |  |
| L angular gyrus/ L middle occipital gyrus/ L middle temporal gyrus/ L inferior parietal gyrus ***[L MOG]*** | -51 | -66 | 27 | 5.28 | 493* |
| L+R anterior cingulum/ L superior frontal gyrus, medial/ L Superior frontal gyrus, medial orbital/ L superior frontal gyrus ***[arMFC]*** | 3 | 24 | 21 | 4.83 | 272* |
| R middle occipital gyrus/ R angular gyrus/ R middle temporal gyrus/ R inferior temporal gyrus | 45 | -78 | 27 | 4.24 | 199* |
| L+R Precuneus/ R fusiform gyrus/ L+R calcarine gyrus/ L cuneus/ L middle cingulum/ R lingual gyrus ***[PCUN]*** | 3 | -63 | 27 | 4.10 | 433* |
| ***SEED: R olIFG*** |  |  |  |  |  |
|  | **x** | **y** | **Z** | **Z-score (peak voxel)** | **Cluster size (voxel)** |
| ***CSL > TIC (continued)*** |  |  |  |  |  |
| ***SEED: R olIFG*** |  |  |  |  |  |
| L angular gyrus/ L middle occipital gyrus/ L inferior parietal gyrus | -39 | -75 | 45 | 4.12 | 108 |
| ***SEED: L olIFG*** |  |  |  |  |  |
| L angular gyrus/ L middle occipital gyrus /L middle temporal gyrus ***[L MOG]*** | -45 | -78 | 30 | 4.36 | 211* |
| R middle occipital gyrus /R middle temporal gyrus/ R angular gyrus | 51 | -72 | 27 | 3.76 | 83 |
| ***SEED: R pSTS*** |  |  |  |  |  |
| L postcentral gyrus/ L supramarginal gyrus/ L inferior parietal gyrus/ L superior temporal gyrus ***[L SMAR]*** | -39 | -33 | 42 | 5.02 | 337* |
| L inferior frontal gyrus p. opercularis and p. triangularis/ L middle frontal gyrus/ L precentral gyrus | -39 | 12 | 27 | 4.82 | 130* |
| R inferior frontal gyrus p. opercularis and p. triangularis/ R middle frontal gyrus/ R precentral gyrus ***[R pdIFG]*** | 54 | 18 | 30 | 4.31 | 134* |
| R supramarginal gyrus/ R superior temporal gyrus/ R angular gyrus/ R middle temporal gyrus ***[R mSTG]*** | 60 | -48 | 27 | 3.89 | 330* |
| R inferior frontal gyrus p. triangularis and p. opercularis | 54 | 21 | 9 | 3.64 | 55 |
| ***SEED: R MOG*** |  |  |  |  |  |
| R superior temporal gyrus/ R supramarginal gyrus | 69 | -36 | 21 | 3.79 | 73 |
| ***SEED: prMFC*** |  |  |  |  |  |
| L middle temporal gyrus/ L angular gyrus/ L middle occipital gyrus ***[L MOG]*** | -51 | -72 | 24 | 4.29 | 205* |
| R middle temporal gyrus/ R middle occipital gyrus | 51 | -72 | 12 | 3.81 | 53 |
| ***SEED: R FUS*** |  |  |  |  |  |
| R supramarginal gyrus/ R superior temporal gyrus/ R postcentral gyrus | 69 | -21 | 27 | 3.90 | 78 |
| L supramarginal gyrus/ L postcentral gyrus | -57 | -21 | 24 | 3.74 | 54 |
| ***TIC > CSL*** |  |  |  |  |  |
| ***SEED: R LING*** |  |  |  |  |  |
| No cluster above threshold |  |  |  |  |  |
| ***SEED: L LING*** |  |  |  |  |  |
| No cluster above threshold |  |  |  |  |  |
| ***SEED: L MOG*** |  |  |  |  |  |
| R superior parietal gyrus/ R postcentral gyrus | 18 | -51 | 72 | 3.66 | 54 |
| ***SEED: arMFC*** |  |  |  |  |  |
| No cluster above threshold |  |  |  |  |  |
|  | **x** | **y** | **Z** | **Z-score (peak voxel)** | **Cluster size (voxel)** |
| ***TIC > CSL (continued)*** |  |  |  |  |  |
| ***SEED: midCG*** |  |  |  |  |  |
| No cluster above threshold |  |  |  |  |  |
| ***SEED: PCUN*** |  |  |  |  |  |
| No cluster above threshold |  |  |  |  |  |
| ***SEED: R pdIFG*** |  |  |  |  |  |
| R superior temporal gyrus/ R Rolandic operculum/ R Heschl’s gyrus/ R middle temporal gyrus/ R inferior frontal gyrus p. opercularis and p. triangularis/ R supramarginal gyrus ***[R mSTG, R STG/MTG]*** | 66 | 18 | 9 | 5.22 | 1039* |
| L supramarginal gyrus/ L superior temporal gyrus/ L Rolandic operculum/ L postcentral gyrus/ L Heschl’s gyrus ***[L SMAR, L STG/MTG]*** | -60 | -21 | 15 | 4.43 | 780* |
| R+L supplementary motor area ***[SMA]*** | 3 | -3 | 60 | 4.03 | 123* |
| ***SEED: R mSTG*** |  |  |  |  |  |
| R superior temporal gyrus/ R Rolandic operculum/ R Heschl’s gyrus ***[R STG/MTG]*** | 57 | -15 | 9 | 4.64 | 508* |
| L superior temporal gyrus/ L supramarginal gyrus / L Rolandic operculum/ L postcentral gyrus / L Heschl’s gyrus ***[L SMAR, L STG/MTG]*** | -45 | -24 | 15 | 4.29 | 459* |
| R+L supplementary motor area/ R + L middle cingulum/ L+R superior frontal gyrus ***[SMA]*** | -9 | 3 | 57 | 3.98 | 221* |
| ***SEED: L SMAR*** |  |  |  |  |  |
| R superior temporal gyrus/ R Rolandic operculum/ R Heschl’s gyrus/ R middle temporal gyrus ***[R mSTG, R STG/MTG]*** | 48 | -30 | 3 | 5.09 | 700* |
| L superior temporal gyrus/ L Rolandic operculum/ L postcentral gyrus/ L supramarginal gyrus/ L Heschl’s gyrus ***[L STG/MTG]*** | -57 | -15 | 12 | 4.21 | 399* |
| R precentral gyrus/ R inferior frontal gyrus p. opercularis/ R middle frontal gyrus | 51 | 9 | 42 | 3.85 | 100 |
| R+L supplementary motor area/ L middle cingulum | -6 | 9 | 51 | 3.71 | 92 |
| ***SEED: R olIFG*** |  |  |  |  |  |
| R Heschl’s gyrus/ R superior temporal gyrus/ R Rolandic operculum/ R middle temporal gyrus ***[R mSTG, R STG/MTG]*** | 48 | -15 | 6 | 4.90 | 723* |
| L superior temporal gyrus/ L Rolandic operculum/ L postcentral gyrus/ L supramarginal gyrus/ L Heschl’s gyrus ***[L STG/MTG]*** | -54 | -15 | 12 | 4.62 | 535* |
| R inferior frontal gyrus p. triangularis/ R insula | 33 | 24 | 9 | 4.13 | 53 |
|  | **x** | **y** | **Z** | **Z-score (peak voxel)** | **Cluster size (voxel)** |
| ***TIC > CSL (continued)*** |  |  |  |  |  |
| ***SEED: L olIFG*** |  |  |  |  |  |
| R superior temporal gyrus/ R Rolandic operculum/ R Heschl’s gyrus/ R supramarginal gyrus/ R middle temporal gyrus ***[R mSTG, R STG/MTG]*** | 63 | -33 | 12 | 4.18 | 323* |
| R inferior frontal gyrus p. opercularis and p. triangularis/ R precentral gyrus/ R middle frontal gyrus ***[R pdIFG]*** | 51 | 9 | 27 | 4.08 | 138* |
| L superior temporal gyrus/ L Rolandic operculum/ L middle temporal gyrus/ L supramarginal gyrus/ L postcentral gyrus ***[L STG/MTG]*** | -51 | -21 | 3 | 3.83 | 170* |
| ***SEED: R pSTS*** |  |  |  |  |  |
| No cluster above threshold |  |  |  |  |  |
| ***SEED: R MOG*** |  |  |  |  |  |
| L middle frontal gyrus/ L superior frontal gyrus/ L superior frontal gyrus, medial | -21 | 30 | 42 | 4.51 | 120* |
| L+R precuneus/ L+R middle cingulum/ R+L paracentral lobule ***[midCG]*** | 0 | -48 | 57 | 4.21 | 259* |
| ***SEED: prMFC*** |  |  |  |  |  |
| L postcentral gyrus/ L superior temporal gyrus/ L Rolandic operculum/ L supramarginal gyrus ***[L SMAR, L STG/MTG]*** | -57 | -12 | 15 | 4.19 | 226* |
| R inferior frontal gyrus p. opercularis and p. triangularis | 51 | 15 | 18 | 4.12 | 95 |
| R Heschl’s gyrus/ R superior temporal gyrus/ R rolandic operculum/ R supramarginal gyrus ***[R mSTG, R STG/MTG]*** | 51 | -9 | 6 | 4.11 | 434* |
| ***SEED: R FUS*** |  |  |  |  |  |
| No cluster above threshold |  |  |  |  |  |

Activations thresholded at p < 0.001, uncorrected with a cluster size k > 50 voxels. Coordinates refer to the MNI system. * p < 0.05, **FWE** corrected for multiple comparisons across the whole brain **at the cluster level** and Bonferroni-corrected for number of PPI seeds (see Materials and Methods). ROI names in brackets symbolize overlap between significant target clusters in the whole-brain analyses and respective ROIs. **Seed specific cluster size thresholds for a FWE–correction in voxels: R LING: ≥ 63, L LING: ≥ 48, L MOG: ≥ 62, arMFC: ≥ 50, midCG: ≥ 52, PCUN: ≥ 60, R pdIFG: ≥ 60, R mSTG: ≥ 58, L SMAR: ≥ 57, R olIFG: ≥ 62, L olIFG: ≥ 65, R pSTS: ≥ 58, R MOG: ≥ 53, prMFC: ≥ 56, R FUS: ≥ 53.**
