# Supplementary material for: Different Types of Laughter Modulate Connectivity within Distinct Parts of the Laughter Perception Network
Source: PLoS One. 2013 May 8;8(5):e63441. doi: 10.1371/journal.pone.0063441 (PMC3648477; doi:10.1371/journal.pone.0063441)
Supplement: Table S7 — Whole-brain analyses. Relative changes in cerebral functional connectivity (PPI) associated with the perception of different types of complex social laughter (joyful - JOY, taunting - TAU). (DOC) [file pone.0063441.s007.doc]

**Table S7:** Whole-brain analyses: Relative changes in cerebral functional connectivity (PPI) associated with the perception of different types of complex social laughter (joyful - JOY, taunting - TAU).

|  | **x** | **y** | **Z** | **Z-score (peak voxel)** | **Cluster size (voxel)** |
| --- | --- | --- | --- | --- | --- |
| ***JOY > TAU*** |  |  |  |  |  |
| ***SEED: R LING*** |  |  |  |  |  |
| R superior temporal gyrus/ R supramarginal gyrus/ R middle temporal gyrus ***[R STG/MTG]*** | 57 | -42 | 12 | 4.13 | 234* |
| L superior temporal gyrus/ L Heschl’s gyrus/ R Rolandic operculum/ R middle temporal gyrus ***[L SMAR, L STG/MTG]*** | -30 | -27 | 12 | 4.10 | 338* |
| L + R supplementary motor area | -3 | -6 | 63 | 4.03 | 87 |
| ***SEED: L LING -*** No cluster above threshold |  |  |  |  |  |
| ***SEED: L MOG -*** No cluster above threshold |  |  |  |  |  |
| ***SEED: arMFC -*** No cluster above threshold |  |  |  |  |  |
| ***SEED: midCG -*** No cluster above threshold |  |  |  |  |  |
| ***SEED: PCUN*** |  |  |  |  |  |
| R middle temporal gyrus | 48 | -63 | 9 | 4.04 | 53 |
| ***SEED: R pdIFG*** |  |  |  |  |  |
| R superior temporal gyrus/ R middle temporal gyrus ***[R STG/MTG]*** | 66 | -24 | 6 | 4.20 | 221* |
| ***SEED: R mSTG -*** No cluster above threshold |  |  |  |  |  |
| ***SEED: L SMAR -*** No cluster above threshold |  |  |  |  |  |
| ***SEED: R olIFG -*** No cluster above threshold |  |  |  |  |  |
| ***SEED: L olIFG*** |  |  |  |  |  |
| R angular gyrus/ R middle occipital gyrus/ R middle temporal gyrus | 48 | -72 | 33 | 3.83 | 60 |
| ***SEED: R pSTS*** |  |  |  |  |  |
| R precentral gyrus/ R superior temporal gyrus/ R Rolandic operculum/ R supramarginal gyrus/ R Heschl’s gyrus ***[R STG/MTG]*** | 45 | -3 | 27 | 4.43 | 205* |
| R precuneus/ R cuneus/ R superior occipital gyrus | 24 | -54 | 36 | 4.28 | 75 |
| L superior temporal gyrus/ L postcentral gyrus/ L supramarginal gyrus | -60 | -24 | 9 | 3.73 | 76 |
| L+R supplementary motor area/ L+R superior frontal gyrus/ L middle frontal gyrus | -12 | 12 | 63 | 3.66 | 85 |
| ***SEED: R MOG*** |  |  |  |  |  |
| L superior temporal gyrus/ L supramarginal gyrus/ L Rolandic operculum/ L Heschl’s gyrus ***[L SMAR, L STG/MTG]*** | -51 | -18 | 6 | 4.18 | 151* |
| R superior temporal gyrus/ R middle temporal gyrus ***[R STG/MTG]*** | 69 | -30 | 3 | 4.13 | 344* |
|  | **x** | **y** | **Z** | **Z-score (peak voxel)** | **Cluster size (voxel)** |
| ***JOY > TAU (continued)*** |  |  |  |  |  |
| ***SEED: R MOG*** |  |  |  |  |  |
| L+R supplementary motor area | -6 | 3 | 63 | 4.00 | 80 |
| R insula/ R inferior frontal gyrus p. triangularis, p. orbitalis and p. opercularis | 36 | 15 | 3 | 3.95 | 81 |
| R inferior frontal gyrus p. opercularis and p. triangularis/ R precentral gyrus/ R middle frontal gyrus ***[R pdIFG]*** | 51 | 12 | 30 | 3.92 | 116* |
| L inferior frontal gyrus p. triangularis and p. opercularis/ L insula | -39 | 24 | 12 | 3.61 | 57 |
| ***SEED: prMFC -*** No cluster above threshold |  |  |  |  |  |
| ***SEED: R FUS -*** No cluster above threshold |  |  |  |  |  |
| R supramarginal gyrus/ R inferior parietal gyrus/ R superior temporal gyrus | 48 | -42 | 33 | 4.15 | 59 |
| L calcarine gyrus/ L cuneus/ L superior occipital gyrus/ L middle occipital gyrus | -12 | -87 | 12 | 3.69 | 74 |
| ***TAU > JOY*** |  |  |  |  |  |
| ***SEED: R LING -*** No cluster above threshold |  |  |  |  |  |
| ***SEED: L LING -*** No cluster above threshold |  |  |  |  |  |
| ***SEED: L MOG -*** No cluster above threshold |  |  |  |  |  |
| ***SEED: arMFC -*** No cluster above threshold |  |  |  |  |  |
| R superior temporal gyrus/ R Rolandic operculum ***[R mSTG, R STG/MTG]*** | 45 | -36 | 18 | 3.79 | 154* |
| ***SEED: midCG -*** No cluster above threshold |  |  |  |  |  |
| ***SEED: PCUN -*** No cluster above threshold |  |  |  |  |  |
| ***SEED: R pdIFG -*** No cluster above threshold |  |  |  |  |  |
| ***SEED: R mSTG -*** No cluster above threshold |  |  |  |  |  |
| ***SEED: L SMAR*** |  |  |  |  |  |
| R superior temporal gyrus/ R rolandic operculum/ R Heschl’s gyrus/ R postcentral gyrus/ R supramarginal gyrus ***[R STG/MTG]*** | 63 | -18 | 9 | 4.59 | 223* |
| L superior temporal gyrus/ L postcentral gyrus/ L Rolandic operculum/ L supramarginal gyrus/ L Heschl’s gyrus | -54 | -18 | 12 | 3.99 | 60 |
| ***SEED: R olIFG*** |  |  |  |  |  |
| L superior temporal gyrus/ L Rolandic operculum/ L postcentral gyrus/ L Heschl’s Gyrus/ L supramarginal gyrus ***[L SMAR, L STG/MTG]*** | -45 | -33 | 15 | 4.10 | 155* |
| R superior temporal gyrus/ R Rolandic operculum/ R Heschl’s Gyrus/ R middle temporal gyrus ***[R mSTG, R STG/MTG]*** | 63 | -30 | 9 | 3.91 | 189* |
|  | **x** | **y** | **Z** | **Z-score (peak voxel)** | **Cluster size (voxel)** |
| ***TAU > JOY (continued)*** |  |  |  |  |  |
| ***SEED: L olIFG -*** No cluster above threshold |  |  |  |  |  |
| ***SEED: R pSTS -*** No cluster above threshold |  |  |  |  |  |
| ***SEED: R MOG -*** No cluster above threshold |  |  |  |  |  |
| ***SEED: prMFC -*** No cluster above threshold |  |  |  |  |  |
| ***SEED: R FUS -*** No cluster above threshold |  |  |  |  |  |

Activations thresholded at p < 0.001, uncorrected with a cluster size k > 50 voxels. Coordinates refer to the MNI system. * p < 0.05, **FWE** corrected for multiple comparisons across the whole brain **at the cluster level** and Bonferroni-corrected for number of PPI seeds (see Materials and Methods). ROI names in brackets symbolize overlap between significant target clusters in whole-brain analyses and respective ROIs. **Seed specific cluster size thresholds for FWE–correction in voxels: R LING: ≥ 60, L LING: ≥ 58, L MOG: ≥ 61, arMFC: ≥ 54, midCG: ≥ 52, PCUN: ≥ 57, R pdIFG: ≥ 64, R mSTG: ≥ 57, L SMAR: ≥ 49, R olIFG: ≥ 58, L olIFG: ≥ 53, R pSTS: ≥ 57, R MOG: ≥ 47, prMFC: ≥ 54, R FUS: ≥ 53.**
