# Supplementary material for: Different Types of Laughter Modulate Connectivity within Distinct Parts of the Laughter Perception Network
Source: PLoS One. 2013 May 8;8(5):e63441. doi: 10.1371/journal.pone.0063441 (PMC3648477; doi:10.1371/journal.pone.0063441)
Supplement: Table S8 — Whole-brain analyses. Relative changes in cerebral functional connectivity (PPI) associated with explicit evaluation of laughter type (CAT) as compared to laughter bout counting (COU). (DOC) [file pone.0063441.s008.doc]

**Table S8:** Whole-brain analyses: Relative changes in cerebral functional connectivity (PPI) associated with explicit evaluation of laughter type (CAT) as compared to laughter bout counting (COU).

|  | **x** | **y** | **Z** | **Z-score (peak voxel)** | **Cluster size (voxel)** |
| --- | --- | --- | --- | --- | --- |
| ***CAT > COU*** |  |  |  |  |  |
| ***SEED: R LING*** - No cluster above threshold |  |  |  |  |  |
| ***SEED: L LING*** - No cluster above threshold |  |  |  |  |  |
| ***SEED: L MOG*** |  |  |  |  |  |
| R inferior parietal gyrus/ R supramarginal gyrus | 54 | -45 | 45 | 3.79 | 81 |
| ***SEED: arMFC*** - No cluster above threshold |  |  |  |  |  |
| ***SEED: midCG***- No cluster above threshold |  |  |  |  |  |
| ***SEED: PCUN*** - No cluster above threshold |  |  |  |  |  |
| ***SEED: R pdIFG*** - No cluster above threshold |  |  |  |  |  |
| ***SEED: R mSTG*** - No cluster above threshold |  |  |  |  |  |
| ***SEED: L SMAR*** |  |  |  |  |  |
| L superior occipital gyrus/ L middle occipital gyrus | -24 | -15 | 9 | 4.22 | 79 |
| L Putamen/ L insula/ L Thalamus/ L Heschl’s gyrus/ L Pallidum | -15 | -90 | 21 | 3.72 | 55 |
| ***SEED: R olIFG*** - No cluster above threshold |  |  |  |  |  |
| ***SEED: L olIFG*** - No cluster above threshold |  |  |  |  |  |
| ***SEED: R pSTS*** - No cluster above threshold |  |  |  |  |  |
| ***SEED: R MOG*** - No cluster above threshold |  |  |  |  |  |
| ***SEED: prMFC*** - No cluster above threshold |  |  |  |  |  |
| ***SEED: R FUS*** - No cluster above threshold |  |  |  |  |  |
| ***COU > CAT*** |  |  |  |  |  |
| ***SEED: R LING*** - No cluster above threshold |  |  |  |  |  |
| ***SEED: L LING*** - No cluster above threshold |  |  |  |  |  |

|  | **x** | **y** | **Z** | **Z-score (peak voxel)** | **Cluster size (voxel)** |
| --- | --- | --- | --- | --- | --- |
| ***COU > CAT (continued)*** |  |  |  |  |  |
| ***SEED: L MOG*** - No cluster above threshold |  |  |  |  |  |
| ***SEED: arMFC*** - No cluster above threshold |  |  |  |  |  |
| ***SEED: midCG*** - No cluster above threshold |  |  |  |  |  |
| ***SEED: PCUN*** - No cluster above threshold |  |  |  |  |  |
| ***SEED: R pdIFG*** - No cluster above threshold |  |  |  |  |  |
| ***SEED: R mSTG*** |  |  |  |  |  |
| R inferior parietal gyrus/ R superior parietal gyrus | 48 | -42 | 57 | 4.51 | 92 |
| ***SEED: L SMAR*** - No cluster above threshold |  |  |  |  |  |
| ***SEED: R olIFG*** - No cluster above threshold |  |  |  |  |  |
| ***SEED: L olIFG*** |  |  |  |  |  |
| L anterior cingulum/ R+L superior frontal gyrus, medial/ R middle cingulum | -9 | 27 | 24 | 4.42 | 52 |
| ***SEED: R pSTS*** - No cluster above threshold |  |  |  |  |  |
| ***SEED: R MOG*** - No cluster above threshold |  |  |  |  |  |
| ***SEED: prMFC*** - No cluster above threshold |  |  |  |  |  |
| ***SEED: R FUS*** - No cluster above threshold |  |  |  |  |  |

Activations thresholded at p < 0.001, uncorrected with a cluster size k > 50 voxels. Coordinates refer to the MNI system. **Seed specific cluster size thresholds for FWE–correction at the cluster level in voxels: R LING: ≥ 48, L LING: ≥ 52, L MOG: ≥ 49, arMFC: ≥ 56, midCG: ≥ 56, PCUN: ≥ 46, R pdIFG: ≥ 58, R mSTG: ≥ 49, L SMAR: ≥ 52, R olIFG: ≥ 53, L olIFG: ≥ 50, R pSTS: ≥ 54, R MOG: ≥ 54, prMFC: ≥ 55, R FUS: ≥ 52.**
