# Supplementary material for: Different Types of Laughter Modulate Connectivity within Distinct Parts of the Laughter Perception Network
Source: PLoS One. 2013 May 8;8(5):e63441. doi: 10.1371/journal.pone.0063441 (PMC3648477; doi:10.1371/journal.pone.0063441)
Supplement: Table S9 — ROI analysis of the bilateral amygdalae. Relative changes in cerebral functional connectivity (PPI) following the perception of complex social laughter types (CSL), reflex-like tickling laughter (TIC), different complex social laughter types (JOY, TAU) and explicit versus implicit evaluation of laughter type (CAT,COU). (DOC) [file pone.0063441.s009.doc]

**Table S9:** ROI analysis of the bilateral amygdalae: Relative changes in cerebral functional connectivity (PPI) following the perception of complex social laughter types (CSL), reflex-like tickling laughter (TIC), different complex social laughter types (JOY, TAU) and explicit versus implicit evaluation of laughter type (CAT,COU).

|  | **x** | **y** | **z** | **Z-score (peak voxel)** | **Cluster size (voxel)** |
| --- | --- | --- | --- | --- | --- |
| ***CSL > TIC*** |  |  |  |  |  |
| ***SEED: R LING -*** No cluster above threshold |  |  |  |  |  |
| ***SEED: L LING -*** No cluster above threshold |  |  |  |  |  |
| ***SEED: L MOG -*** No cluster above threshold |  |  |  |  |  |
| ***SEED: arMFC -*** No cluster above threshold |  |  |  |  |  |
| ***SEED: midCG -*** No cluster above threshold |  |  |  |  |  |
| ***SEED: PCUN -*** No cluster above threshold |  |  |  |  |  |
| ***SEED: R pdIFG -*** No cluster above threshold |  |  |  |  |  |
| ***SEED: R mSTG -*** No cluster above threshold |  |  |  |  |  |
| ***SEED: L SMAR -*** No cluster above threshold |  |  |  |  |  |
| ***SEED: R olIFG -*** No cluster above threshold |  |  |  |  |  |
| ***SEED: L olIFG -*** No cluster above threshold |  |  |  |  |  |
| ***SEED: R pSTS -*** No cluster above threshold |  |  |  |  |  |
| ***SEED: R MOG -*** No cluster above threshold |  |  |  |  |  |
| ***SEED: prMFC -*** No cluster above threshold |  |  |  |  |  |
| ***SEED: R FUS -*** No cluster above threshold |  |  |  |  |  |
| ***TIC > CSL*** |  |  |  |  |  |
| ***SEED: R LING -*** No cluster above threshold |  |  |  |  |  |
| ***SEED: L LING -*** No cluster above threshold |  |  |  |  |  |
| ***SEED: L MOG -*** No cluster above threshold |  |  |  |  |  |
| ***SEED: arMFC -*** No cluster above threshold |  |  |  |  |  |
| ***SEED: midCG -*** No cluster above threshold |  |  |  |  |  |
| ***SEED: PCUN -*** No cluster above threshold |  |  |  |  |  |
| ***SEED: R pdIFG -*** No cluster above threshold |  |  |  |  |  |
| ***SEED: R mSTG -*** No cluster above threshold |  |  |  |  |  |
| ***SEED: L SMAR -*** No cluster above threshold |  |  |  |  |  |
| ***SEED: R olIFG -*** No cluster above threshold |  |  |  |  |  |
| ***SEED: L olIFG -*** No cluster above threshold |  |  |  |  |  |
| ***SEED: R pSTS -*** No cluster above threshold |  |  |  |  |  |
| ***SEED: R MOG -*** No cluster above threshold |  |  |  |  |  |
| ***SEED: prMFC -*** No cluster above threshold |  |  |  |  |  |
| ***SEED: R FUS -*** No cluster above threshold |  |  |  |  |  |
| ***HAP > TAU*** |  |  |  |  |  |
| ***SEED: R LING -*** No cluster above threshold |  |  |  |  |  |
| ***SEED: L LING -*** No cluster above threshold |  |  |  |  |  |
| ***SEED: L MOG -*** No cluster above threshold |  |  |  |  |  |
| ***SEED: arMFC -*** No cluster above threshold |  |  |  |  |  |
| ***SEED: midCG -*** No cluster above threshold |  |  |  |  |  |
| ***SEED: PCUN:*** R Amygdala | 30 | -3 | -15 | 2.88 | 8 |
| L Amygdala | - | - | - | - | - |
| ***SEED: PCUN -*** No cluster above threshold |  |  |  |  |  |
| ***SEED: R pdIFG -*** No cluster above threshold |  |  |  |  |  |
|  | **x** | **y** | **z** | **Z-score (peak voxel)** | **Cluster size (voxel)** |
| ***HAP > TAU (continued)*** |  |  |  |  |  |
| ***SEED: R mSTG:*** R Amygdala | - | - | - | - | - |
| L Amygdala | -24 | 3 | -18 | 3.15 | 25 |
| ***SEED: R mSTG -*** No cluster above threshold |  |  |  |  |  |
| ***SEED: L SMAR -*** No cluster above threshold |  |  |  |  |  |
| ***SEED: R olIFG -*** No cluster above threshold |  |  |  |  |  |
| ***SEED: L olIFG -*** No cluster above threshold |  |  |  |  |  |
| ***SEED: R pSTS -*** No cluster above threshold |  |  |  |  |  |
| ***SEED: R MOG -*** No cluster above threshold |  |  |  |  |  |
| ***SEED: prMFC -*** No cluster above threshold |  |  |  |  |  |
| ***SEED: R FUS -*** No cluster above threshold |  |  |  |  |  |
| ***TAU > HAP*** |  |  |  |  |  |
| ***SEED: R LING -*** No cluster above threshold |  |  |  |  |  |
| ***SEED: L LING -*** No cluster above threshold |  |  |  |  |  |
| ***SEED: L MOG -*** No cluster above threshold |  |  |  |  |  |
| ***SEED: arMFC -*** No cluster above threshold |  |  |  |  |  |
| ***SEED: midCG -*** No cluster above threshold |  |  |  |  |  |
| ***SEED: PCUN -*** No cluster above threshold |  |  |  |  |  |
| ***SEED: R pdIFG:*** R Amygdala | 30 | -3 | -21 | 2.86 | 4 |
| L Amygdala | - | - | - | - | - |
| ***SEED: R pdIFG -*** No cluster above threshold |  |  |  |  |  |
| ***SEED: R mSTG -*** No cluster above threshold |  |  |  |  |  |
| ***SEED: L SMAR -*** No cluster above threshold |  |  |  |  |  |
| ***SEED: R olIFG -*** No cluster above threshold |  |  |  |  |  |
| ***SEED: L olIFG -*** No cluster above threshold |  |  |  |  |  |
| ***SEED: R pSTS -*** No cluster above threshold |  |  |  |  |  |
| ***SEED: R MOG -*** No cluster above threshold |  |  |  |  |  |
| ***SEED: prMFC -*** No cluster above threshold |  |  |  |  |  |
| ***SEED: R FUS -*** No cluster above threshold |  |  |  |  |  |
| ***CAT > COU*** |  |  |  |  |  |
| ***SEED: R LING -*** No cluster above threshold |  |  |  |  |  |
| ***SEED: L LING -*** No cluster above threshold |  |  |  |  |  |
| ***SEED: L MOG:*** R Amygdala | - | - | - | - | - |
| L Amygdala | -27 | -9 | -12 | 3.05 | 11 |
| ***SEED: arMFC -*** No cluster above threshold |  |  |  |  |  |
| ***SEED: midCG -*** No cluster above threshold |  |  |  |  |  |
| ***SEED: PCUN -*** No cluster above threshold |  |  |  |  |  |
| ***SEED: R pdIFG -*** No cluster above threshold |  |  |  |  |  |
| ***SEED: R mSTG:*** R Amygdala | 30 | -6 | -12 | 2.94 | 14 |
| L Amygdala | -18 | -6 | -15 | 2.50 | 3 |
| ***SEED: L SMAR:*** R Amygdala | 24 | -3 | 15 | 3.40 | 24 |
| L Amygdala | -24 | -6 | -15 | 2.62 | 10 |
| ***SEED: R olIFG -*** No cluster above threshold |  |  |  |  |  |
| ***SEED: L olIFG -*** No cluster above threshold |  |  |  |  |  |
| ***SEED: R pSTS:*** R Amygdala | - | - | - | - | - |
| L Amygdala | -27 | -3 | -12 | 2.57 | 3 |
|  | **x** | **y** | **z** | **Z-score (peak voxel)** | **Cluster size (voxel)** |
| ***CAT > COU (continued)*** |  |  |  |  |  |
| ***SEED: R pSTS:*** L Amygdala (continued) | -24 | -3 | -21 | 2.55 | 4 |
| ***SEED: R MOG -*** No cluster above threshold |  |  |  |  |  |
| ***SEED: prMFC -*** No cluster above threshold |  |  |  |  |  |
| ***SEED: R FUS -*** No cluster above threshold |  |  |  |  |  |
| ***COU > CAT*** |  |  |  |  |  |
| ***SEED: R LING -*** No cluster above threshold |  |  |  |  |  |
| ***SEED: L LING -*** No cluster above threshold |  |  |  |  |  |
| ***SEED: L MOG -*** No cluster above threshold |  |  |  |  |  |
| ***SEED: arMFC -*** No cluster above threshold |  |  |  |  |  |
| ***SEED: midCG -*** No cluster above threshold |  |  |  |  |  |
| ***SEED: PCUN -*** No cluster above threshold |  |  |  |  |  |
| ***SEED: R pdIFG -*** No cluster above threshold |  |  |  |  |  |
| ***SEED: R mSTG -*** No cluster above threshold |  |  |  |  |  |
| ***SEED: L SMAR -*** No cluster above threshold |  |  |  |  |  |
| ***SEED: R olIFG -*** No cluster above threshold |  |  |  |  |  |
| ***SEED: L olIFG -*** No cluster above threshold |  |  |  |  |  |
| ***SEED: R pSTS -*** No cluster above threshold |  |  |  |  |  |
| ***SEED: R MOG -*** No cluster above threshold |  |  |  |  |  |
| ***SEED: prMFC -*** No cluster above threshold |  |  |  |  |  |
| ***SEED: R FUS -*** No cluster above threshold |  |  |  |  |  |

Activations thresholded at p < 0.01, uncorrected with a cluster size k ≥ 3 voxels. Coordinates refer to the MNI system.
